# Humoral and cell-mediated immune responses to COVID-19 vaccines up to 6 months post three-dose primary series in adults with inborn errors of immunity and their breakthrough infections

**DOI:** 10.3389/fimmu.2024.1501908

**Published:** 2025-01-21

**Authors:** Dana Unninayar, Emilia L. Falcone, Hugo Chapdelaine, Donald C. Vinh, Karina A. Top, Beata Derfalvi, Thomas B. Issekutz, Hélène Decaluwe, Anne Pham-Huy, Julia Upton, Stephen D. Betschel, Tamar Rubin, Sneha Suresh, Nicola A. M. Wright, Luis Murguía-Favela, Tatiana Kalashnikova, Lisa Barrett, Sharon Oldford, Marc-Andre Langlois, Corey Arnold, Manish Sadarangani, Tinghua Zhang, Tim Ramsay, Dina Yazji, Juthaporn Cowan

**Affiliations:** ^1^ Inflammation and Chronic Disease Program, The Ottawa Hospital Research Institute, Ottawa, ON, Canada; ^2^ Department of Medicine, Centre Hospitalier de l’Université de Montréal, Montreal, QC, Canada; ^3^ Department of Medicine, McGill University Health Centre, Montreal, QC, Canada; ^4^ IWK Health, Department of Pediatrics, Dalhousie University, Halifax, NS, Canada; ^5^ Centre Hospitalier de l’Université (CHU) Ste Justine, Centre de Recherche, Montreal, QC, Canada; ^6^ Department of Pediatrics, Children’s Hospital of Eastern Ontario, Ottawa, ON, Canada; ^7^ The Hospital for Sick Children, Department of Pediatrics, University of Toronto, Toronto, ON, Canada; ^8^ Clinical Immunology and Allergy, Unity Health Toronto, Toronto, ON, Canada; ^9^ Department of Pediatrics and Child Health, University of Manitoba, Winnipeg, MB, Canada; ^10^ Department of Pediatrics, University of Alberta, Edmonton, AB, Canada; ^11^ Alberta Children’s Hospital, Calgary, AB, Canada; ^12^ Department of Medicine, Dalhousie University, Halifax, NS, Canada; ^13^ Department of Biochemistry, Microbiology, and Immunology, University of Ottawa, Ottawa, ON, Canada; ^14^ Department of Pediatrics, University of British Columbia, Vancouver, BC, Canada

**Keywords:** COVID-19, vaccine response, inborn error of immunity, humoral immunity, cellular mediated immunity, immunogenicity

## Abstract

**Purpose:**

Many individuals with inborn errors of immunity (IEIs) have poor humoral immune (HI) vaccine responses. Only a few studies have examined specific cell-mediated immune (CMI) responses to coronavirus disease 2019 (COVID-19) vaccines in this population. Therefore, the purpose of this study was to examine HI and CMI responses up to 6 months post-COVID-19 vaccine dose 3 in adults with IEIs.

**Methods:**

A multi-center prospective observational study was conducted across Canada to collect severe acute respiratory syndrome coronavirus-2 (SARS-CoV-2)-specific HI and CMI data at 4- and 24-week intervals after vaccine doses 2 and 3 (D2 + 4wk/D2 + 24wk/D3 + 4wk/D3 + 24wk).

**Results:**

A total of 149 adults with IEIs and 423 healthy controls were recruited from July 2021 to October 2023. Geometric mean anti-spike IgG (binding antibody units/mL) and spike-specific T-cell responses [IFN-γ^+^ T cells/10^6^ peripheral blood mononuclear cells (PBMCs)] were significantly lower in IEIs compared to controls at D2 + 4wk, D3 + 4wk, and D3 + 24wk. However, at 6 months after completing the primary series (three doses for IEIs and two doses for healthy), both HI and CMI responses of both IEI participants and healthy controls persisted and were comparable. There was a strong correlation between neutralizing antibody titer (ID50) and anti-spike IgG but not between ID50 and CMI. There was only one reported case of hospitalized COVID-19 disease before and none after completing the primary series among IEI participants.

**Conclusion:**

Adults with IEIs mounted both HI and CMI responses following COVID-19 vaccines, which were lower than those of healthy individuals but were present at least up to 6 months after dose 3. These data support the initial recommendation for a three-dose primary series among IEIs.

## Introduction

Individuals with inborn errors of immunity (IEIs) have germline variants in single genes that lead to defects in the innate or adaptive immune system. IEIs are classified by the branch of the immune system affected: immunodeficiencies affecting cellular and humoral immunity, predominantly antibody deficiencies, congenital defects of phagocytes, and complement deficiencies, as well as other defects in intrinsic and innate immunity. The spectrum of diseases included in IEIs is an ever-growing area of clinical research and also includes diseases of immune dysregulation, autoinflammatory disorders, and bone marrow failure. Many individuals with IEIs are at increased risk of certain types of infections but may also present with features of autoimmunity and malignancy and other features of immune dysregulation. Individuals with IEIs have been found to be at increased risk of severe coronavirus disease 2019 (COVID-19), with higher rates of hospitalization, intensive care unit (ICU) admission, and death compared to the general population (1–4). Further, several case reports and case series have described an increased frequency of protracted COVID-19, prolonged viral replication, and variant evolution in patients with immunodeficiencies (5–14).

COVID-19 vaccines are one of the most effective public health strategies to reduce morbidity and mortality from COVID-19 (15–18). Studies have demonstrated that individuals with IEIs often have suboptimal immune responses to vaccines, and therefore, additional vaccine doses may be warranted (19). Most immunization advisory groups initially recommended a three-dose COVID-19 primary series for individuals with immunodeficiencies as compared to two doses for healthy individuals (20–22). However, few studies have compared the responses, especially cell-mediated immune (CMI) responses, between individuals with IEIs and healthy individuals after dose 3 (23).

The primary objective of this study was to longitudinally assess both humoral immune (HI) and CMI responses to COVID-19 vaccines available in Canada [BNT162b2 (Pfizer-BioNTech), mRNA-1273 (Moderna), or ChAdOx1 nCoV-19 (AstraZeneca)] in patients with IEIs versus healthy individuals up to 6 months following their primary vaccination series. The secondary objective was to measure the frequency and severity of COVID-19 infections post-vaccination and vaccine safety among these patients.

## Materials and methods

### Study design and participants

A multi-center prospective observational cohort study was conducted between July 2021 and March 2024 at seven adult and seven pediatric centers to assess COVID-19 vaccination immunogenicity and safety in IEIs. The study was conducted in accordance with the Declaration of Helsinki. The Ottawa Hospital Research Institute was the lead site and obtained overarching ethics approval (CTO#1978) for Ontario sites. Each study site also obtained approval from their local ethics board prior to study commencement. All participants provided written informed consent or assent before enrollment. There was no restriction on the number of COVID-19 vaccine doses received prior to study enrolment if they were less than 24 weeks after dose 4 and received any of the following vaccines available in Canada: BNT162b2, mRNA-1273, or ChAdOx1 nCoV-19.

The current article reports data on adult participants aged 18 years or older and up to 24 weeks after dose 3. Eligible participants with IEIs were recruited into three study subgroups based on the type of IEIs (24): A) predominant antibody deficiency (PAD), B) immunodeficiencies affecting cellular and humoral immunity [combined B-cell and T-cell immunodeficiency (CID)], and C) defects of intrinsic and innate immunity (III) or other IEIs not meeting criterion A) or B). Healthy individuals who did not report any known immunocompromising conditions at the time of initial study screening were recruited as controls. Additional healthy controls meeting our eligibility criteria were recruited from the Stop the Spread Ottawa study, a prospective cohort study investigating longitudinal antibody titers since October 2020 (25, 26).

Participants were excluded if they had been diagnosed with an IgG subclass deficiency without evidence of functional antibody deficiency and isolated IgA deficiency, had evidence of human immunodeficiency virus infection, had a positive COVID-19 infection by PCR or rapid antigen test (RAT) <12 weeks prior to vaccination, or were otherwise contraindicated from receiving COVID-19 vaccines.

### Vaccination

All participants received vaccines in accordance with their local provincial vaccination guidance, which had varied recommended intervals between vaccine doses. In general, dose 2 was administered 4–12 weeks after dose 1, and dose 3 was administered 6–24 weeks after dose 2.

### Sample collection

The study protocol was initially developed in early 2021 when two COVID-19 vaccine doses were recommended as a primary series. Thus, we collected blood at 0–28 days prior to administration of dose 1 (D1) as well as 0–14 days before dose 2 (D2), 4 ± 1 weeks after (D2 + 4wk), and 24 ± 4 weeks after (D2 + 24wk) dose 2. A three-dose primary series was recommended for IEIs as of September 2021 in Canada (27). The study protocol was therefore amended to include data collection before and after dose 3 for IEIs and controls. To minimize the frequency of blood draws and maintain participant retention, we omitted the study visit at D2 + 4wk but added 0–14 days before dose 3 (D3), as well as 4 ± 1 (D3 + 4wk) and 24 ± 4 (D3 + 24wk) weeks after dose 3. Participants who did not want to proceed with dose 3 despite recommendations were followed up until 48 ± 4 weeks after dose 2 (D2 + 48wk) for blood collection.

Plasma and peripheral blood mononuclear cell (PBMC) samples were isolated from participants’ blood and stored at each participating center prior to shipment to the University of Ottawa Serology and Diagnostics High Throughput Facility (plasma) for antibody assays and Dalhousie University (PBMC) for T-cell IFN-γ enzyme-linked immunosorbent spot (ELISpot) assay.

### Immunogenicity

Plasma samples were assessed for levels of IgG, IgA, and IgM antibodies against severe acute respiratory syndrome coronavirus-2 (SARS-CoV-2) trimeric spike (S), receptor binding domain of spike (RBD), and nucleoprotein (N) by chemiluminescent ELISA (28). Antibody neutralization was assessed using surrogate neutralization ELISA on the full trimeric spike (wild-type and B.1.1.529-Omicron BA.1 subvariant, NRC Metrology and laboratory of Dr. Yves Durocher) (26). IgG serological titers were reported in WHO international units as binding antibody units (BAU)/mL. IgA and IgM titers were reported as concentrations compared to laboratory-calibrated standard curves (ng/μL). Neutralization data were reported as the inhibitory dilution at 50% inhibition (ID50) calculated from a four-parameter logarithmic regression applied to a 5-point serial dilution of samples.

Human IFN-γ ELISPOT set (BD Biosciences, Mississauga, ON, Canada) was used as per manufacturer instructions to determine the proportion of SARS-CoV-2-specific T cells. PBMCs were assayed in duplicate at 2 × 10^5^/well after 18-hour stimulation with 2.5 µg/mL phytohemagglutinin-L (PHA; Life Technologies, Burlington, ON, Canada) as the positive control, an equivalent concentration of dimethyl sulfoxide (DMSO) (Sigma-Aldrich, St. Louis, MO, USA) as the negative control (0.32%), or 1 µg/mL peptide pools, consisting mainly of 15 mer sequences with 11 amino acids overlapping, derived from the complete protein-coding sequences of S-protein from wild-type, B.1.617.2-Delta (S-Delta) and B.1.1.529-Omicron (S-Omicron) variants, wild-type N, or control peptide pool. Spots were enumerated using a CTL-ImmunoSpot^®^ S6 Micro Analyzer (CTL, Cleveland, OH, USA), and CMI response was reported as the proportion of SARS-CoV-2 specific T cells (IFN-γ^+^ T cells/10^6^ PBMCs).

### Vaccine safety and breakthrough COVID-19 infection

Vaccine safety questionnaires were completed by the participants 7 and 28 days after each dose and 24 weeks after their last dose. Details on medically attended adverse events (MAAEs) including onset, symptoms, type of medical attention sought, treatments, severity (29), and evaluation of vaccine relatedness were collected. Additionally, information on historical COVID-19 infections was collected at the baseline visit. Breakthrough COVID-19 infections confirmed by PCR or RAT were noted at each follow-up.

### Analysis

Baseline demographics were summarized using descriptive analyses. Age, body mass index (BMI), and baseline laboratory parameters were summarized using means and standard deviations. Intervals in days between vaccine doses were reported as median with interquartile ranges (IQRs). The remainder of the demographic data were expressed as percentages. Primary analyses were conducted by comparing HI and CMI responses of IEIs with those of controls at each timepoint and 4 weeks after completing the primary series. Secondary analyses included the comparison of CMI and HI responses based on the type of IEIs. HI responses were stratified and analyzed by use of immunoglobulin replacement therapy (IGRT) and previous COVID-19 infection confirmed by PCR or RAT. Hybrid immunity was defined as having positive anti-N IgG and/or previous SARS-CoV-2 infection. Confidence intervals of geometric mean values of serological titers and IFN-γ^+^ T cells/10^6^ PBMCs were calculated. The independent t-test was used to compare geometric means at each timepoint between IEIs and controls. The proportion (Fisher’s exact test) of COVID-19 infections post-vaccination was compared between participant groups and IEI subgroups. Geometric means of HI and CMI among IEI subgroups and controls were compared using one-way ANOVA. The Mann–Whitney U-test was used to compare medians between IEIs and controls. The Kruskal–Wallis test was used to compare medians between IEI subgroups and controls. All tests were two-sided with statistical significance set at α = 0.05. Analyses were performed using SAS version 9.4 (SAS Institute Inc., Cary, NC, USA), and graphs were generated using Prism version 10.2 (GraphPad Software, LLC, Boston, MA, USA).

## Results

A total of 149 adult participants with IEIs (99 in the PAD subgroup, 35 in the CID subgroup, and 15 in the III subgroup), 37 controls, and 386 controls from the Stop the Spread Ottawa (SSO) study were recruited (**Supplementary Material**). Participant demographic information is summarized in **Table 1**. The average (SD) age of adult participants with IEIs, our controls, and SSO controls were 47.7 (15.1), 52.5 (13.2), and 47.3 (13.9) years, respectively; 68.1% and 56.5% of our and the SSO controls, respectively, were female, and 57.7% of participants with IEIs were female. The most common IEI was common variable immunodeficiency (CVID) (n = 77). Among the remainder of the participants with PAD, five had agammaglobulinemia, four had hyper-IgM syndrome, five had specific antibody deficiency, one had hypogammaglobulinemia, and seven had other types of PAD. Among CID participants, one had severe combined immunodeficiency (SCID) status post-hematopoietic stem cell transplant, three had CID with syndromic features, two had purine nucleoside phosphorylase (PNP) deficiency, three had 22q11 deletion syndrome, and 25 had other types of CID. Finally, among participants in the III subgroup, two had a diagnosis of chronic granulomatous disease or phagocyte defect, six had complement deficiency, three had autoinflammatory disorders, and four had diseases of immune dysregulation (**Table 1**). A total of 107 of the participants with IEIs were treated with IGRT.

**Table 1:** Baseline demographic data.

	Healthy (N = 37)	SOS healthy cohort (N = 386)	Inborn error of immunity^a^ (N = 149)			
			All (N = 149)	A (N = 99)	B (N = 35)	C (N = 15)
Sociodemographic and health variables						
Mean age in years (SD)	47.3 ± 13.9	52.5 ± 13.2	47.7 ± 15.1	51 ± 14.6	38.6 ± 14.6	47.1 ± 11.4
Female sex at birth, n (%)	288 (68.1%)	21 (56.8%)	86 (57.7%)	62 (62.6%)	17 (48.6%)	7 (46.7%)
BMI (kg/m^2^)	28.3 ± 6.9	No data	27.0 ± 6.2	27.8 ± 6	25.1 ± 5.9	26.1 ± 8
Ethnicity or race, n (%)						
White	383 (90.5%)	33 (89.3%)	133 (89.3%)	90 (90.9%)	32 (91.4%)	11 (73.3%)
South Asian	6 (1.42%)	0	0	0	0	0
East Asian	3 (0.7%)	1 (2.7%)	3 (2.0%)	1 (1.0%)	1 (2.9%)	1 (6.7%)
Latin American	1 (0.2%)	0	2 (1.3%)	1 (1.0%)	0 (0%)	1 (6.7%)
Southeast Asian	6 (1.4%)	0	1 (0.7%)	0	0	1 (6.7%)
Arab/West Asian	19 (4.3%)	1 (2.7%)	3 (2.0%)	2 (2.0%)	0	1 (6.7%)
Black	3 (0.7%)	3 (0.7%)	0	0	0	0
Prefer to self-describe	2 (0.5%)	2 (5.4%)	4 (2.7%)	3 (3.0%)	0	1 (6.7%)
Prefer not to answer	0	0	3 (2.0%)	2 (2.0%)	1 (2.9%)	0
Immunoglobulin treatment						
Subcutaneous immunoglobulin (SCIG)	0	0	83	64	18	1
Intravenous immunoglobulin (IVIG)	0	0	24	20	3	1
Immunosuppressive agents						
Current biological therapy	0	0	6	1	2	3
Other immunosuppressants	0	0	2	1	0	1
Comorbidities						
Thrombocytopenia	0	No data	16 (10.7%)	8 (8.1%)	5 (14.3%)	3 (20.0%)
Leukopenia	0		9 (6.0%)	6 (6.1%)	1 (2.9%)	2 (13.3%)
Lymphopenia	0		9 (6.0%)	3 (3.0%)	5 (14.3%)	1 (6.7%)
Neutropenia	0		3 (2.0%)	2 (2.0%)	0	1 (6.7%)
Solid organ transplant	1		3	2	1	0
Blood/bone marrow transplant	0		4	0	4	0
Cancer	0		7 (4.7%)	5 (5.1%)	2 (5.7%)	0
Asthma	5 (13.5%)		49 (32.9%)	32 (32.3%)	14 40.0%)	3 (20.0%)
COPD	0		4 (2.7%)	2 (2.0%)	2 (5.7%)	0
Other respiratory disease	0		26 (17.5%)	17 (17.2%)	6 (17.1%)	3 (20.0%)
Congestive heart failure	1 (2.7%)		1 (0.7%)	0	1 (2.9%)	0
Chronic kidney disease	0		9 (6.04%)	3 (3.03%)	3 (8.6%)	3 (20.0%)
Chronic liver disease	2 (5.4%)		9 (6.0%)	4 (4.0%)	3 (8.6%)	2 (13.3%)
Chronic neurological disorder	4 (10.8%)		20 (13.4%)	13 (13.1%)	6 (17.1%)	1 (6.7%)
Diabetes type I	1 (2.7%)		4 (2.7%)	3 (3.0%)	0	1 (6.7%)
Diabetes type II	0		3 (2.0%)	1 (1.01%)	1 (2.9%)	1 (6.7%)
Hypertension	4 (10.8%)		23 (15.4%)	17 (17.2%)	4 (11.4%)	2 (13.3%)
Stroke	1 (2.7%)		5 (3.5%)	2 (2.0%)	3 (8.6%)	0
Obesity (BMI ≥ 30)	12 (32.4%)		38 (25.9%)	28 (28.9%)	7 (20.0%)	3 (20.0%)
Median interval in days between vaccine doses (IQR)						
Between doses 1 and 2	80 (61–99)	No data	60.5 (42–72.5)	65 (47–74)	43 (34.5–64)	62 (60–70)
Between doses 2 and 3	184 (179–191.5)		129 (106.5–178)	127 (103–170)	128 (114–186)	161 (97–186)
Baseline laboratory parameters						
WBC (× 10^9^/L)	5.7 ± 1.3		5.5 ± 2.2	5.4 ± 2.5	5.6 ± 1.6	5.7 ± 1.6
Lymphocyte (× 10^6^/L)	605.8 ± 1,000		964.1 ± 824.6	921.9 ± 905.8	993.2 ± 592.4	1,116 ± 864.4
Neutrophil (× 10^6^/L)	1,202 ± 1,835		2,295 ± 1,844	1,875 ± 1,765	3,431 ± 1,743	1,970 ± 1,501
CD4 cell count (× 10^6^/L)	No data		3.7 ± 14.4	5.3 ± 17.4	0.4 ± 0.2	0.2
CD8 cell count (× 10^6^/L)	No data		1.4 ± 4.6	1.8 ± 5.5	0.5 ± 0.4	0.2
IgG (g/L)	9.9 ± 1.6		10.1 ± 3.5	9.9 ± 3.2	9.7 ± 3.4	12.7 ± 4.8
IgA (g/L)	1.8 ± 0.6		0.8 ± 1.0	0.5 ± 0.7	1.1 ± 1.0	1.9 ± 1.3
IgM (g/L)	1.1 ± 0.5		0.7 ± 1.5	0.6 ± 1.7	1.0 ± 1.0	0.8 ± 0.5

BMI, body mass index; COPD, chronic obstructive pulmonary disease; WBC, white blood cell; IQR, interquartile range.

a Type of inborn error of immunity: predominant antibody deficiency (PAD) subgroup—agammaglobulinemia 5 (5.1%), common variable immunodeficiency 77 (77.8%), hyper-IgM syndrome 4 (4.0%); specific antibody deficiency 5 (5.1%), hypogammaglobulinemia 1 (1.0%), and other types of PAD 7 (7.1%); combined immunodeficiency (CID) subgroup—severe combined immunodeficiency status post-hematopoietic stem cell transplantation 1 (5.7%), CID with syndromic features 3 (8.6%), purine nucleoside phosphorylase (PNP) deficiency 2 (5.7%), 22q11 deletion syndrome 3 (8.6%), and other types of CID 25 (71.4%); defects in intrinsic and innate immunity and other inborn errors of immunity (III) subgroup—chronic granulomatous disease or phagocyte defect 2 (13.3%), complement deficiency 6 (40.0%), autoinflammatory disorder 3 (20.0%), and diseases of immune dysregulation 4 (26.7%).

At the time of study enrolment, 20 IEI participants and eight controls were unvaccinated. Of the 129 vaccinated IEI participants, 60 had received three doses, 52 had received two doses, and 16 had received one dose. Out of the 29 vaccinated controls, 12 had received three doses, 15 had received two doses, and two had received one dose (**Table 1**).

### Serological response

At D2 + 4wk, anti-RBD and anti-S IgG titers were significantly lower in participants with IEIs compared to controls {geometric mean (95% CI), 265.5 [95.31–739.7] vs. 4,221.4 [3,436.4–5,185.7] BAU/mL, p < 0.0001; 198.7 [66.43–594.3] vs. 3,830.1 [3,010.6–4,872.7] BAU/mL, p < 0.001, respectively}. However, at 6 months after dose 2 (D2 + 24wk), there was no longer a statistically significant difference in anti-RBD and anti-S IgG levels between IEIs and controls. At D3 + 4wk, anti-RBD and anti-S IgG titers were significantly lower in IEIs compared to controls (834.2 [499.9–1,392.1 vs. 6,961.8 [4,936.2–9,818.7] BAU/mL, p < 0.001; 669.8 [393.9–1,138.8] vs. 5,301.7 [3,402.6–8,260.7] BAU/mL, p < 0.0001, respectively). Anti-RBD and anti-S IgG titers at D3 + 24wk were also significantly lower in IEIs than in controls (852 [428–1,696.1] vs. 3,410.7 [2,664.9–4,365.2], p = 0.0004; 763.6 [402.7–1,447.6] vs. 3,273.7 [2,584.9–4,146], p < 0.0001, respectively).

**Figure 1:**
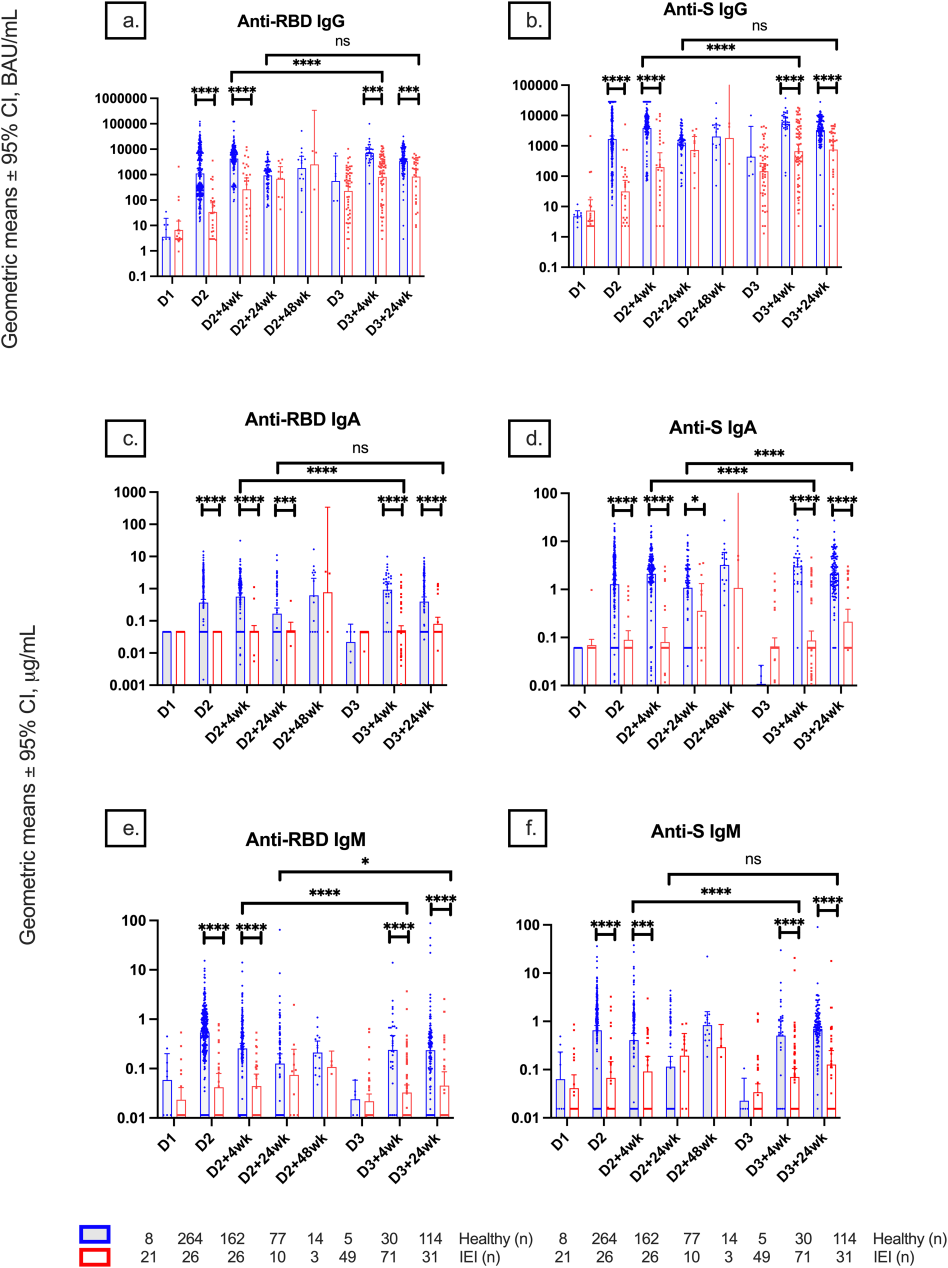
Geometric mean IgG, IgA, and IgM serologies among healthy participants and participants with inborn errors of immunity (IEIs). Geometric mean ± 95% confidence interval of **(A)** anti-RBD IgG, **(B)** anti-S IgG, **(C)** anti-RBD IgA, **(D)** anti-S IgA, **(E)** anti-RBD IgM, and **(F)** anti-S IgM titers among healthy participants and participants with IEIs at different timepoints pre- and post-COVID-19 vaccination up to 24 weeks after dose 3. Number (*n*) of healthy and primary immunodeficient participants with data included per timepoint are indicated. * indicates p < 0.05, *** indicates p < 0.001, **** indicates p < 0.0001, and ns indicates not significant. Error bars indicate the 95% CI. D1, before dose 1; D2, before dose 2; D2 + 4wk, 4 weeks after dose 2; D2 + 24wk, 24 weeks after dose 2; D2 + 48wk, 48 weeks after dose 2; D3, before dose 3; D3 + 4wk, 4 weeks after dose 3; D3 + 24wk, 24 weeks after dose 3; CI, confidence interval; anti-RBD, anti-receptor binding domain; anti-S, anti-spike protein; anti-N, anti-nucleocapsid; ns, not significant.

Comparison of serological responses after completion of the primary series showed lower anti-RBD and anti-S IgG titers in IEIs than controls at 4 weeks but not at 24 weeks (**Figures 1A, B**, **Supplementary Material**). Anti-RBD and anti-S IgA titers at D2, D2 + 4wk, D2 + 24wk, D3 + 4wk, and D3 + 24wk were significantly lower in IEIs compared to controls (**Figures 1C, D**). Anti-RBD and anti-S IgM titers at D2, D2 + 4wk, D3 + 4wk, and D3 + 24wk were also lower in IEIs than in controls (**Figures 1E, F**).

### Evidence of natural infection and hybrid immunity

**Figure 2:**
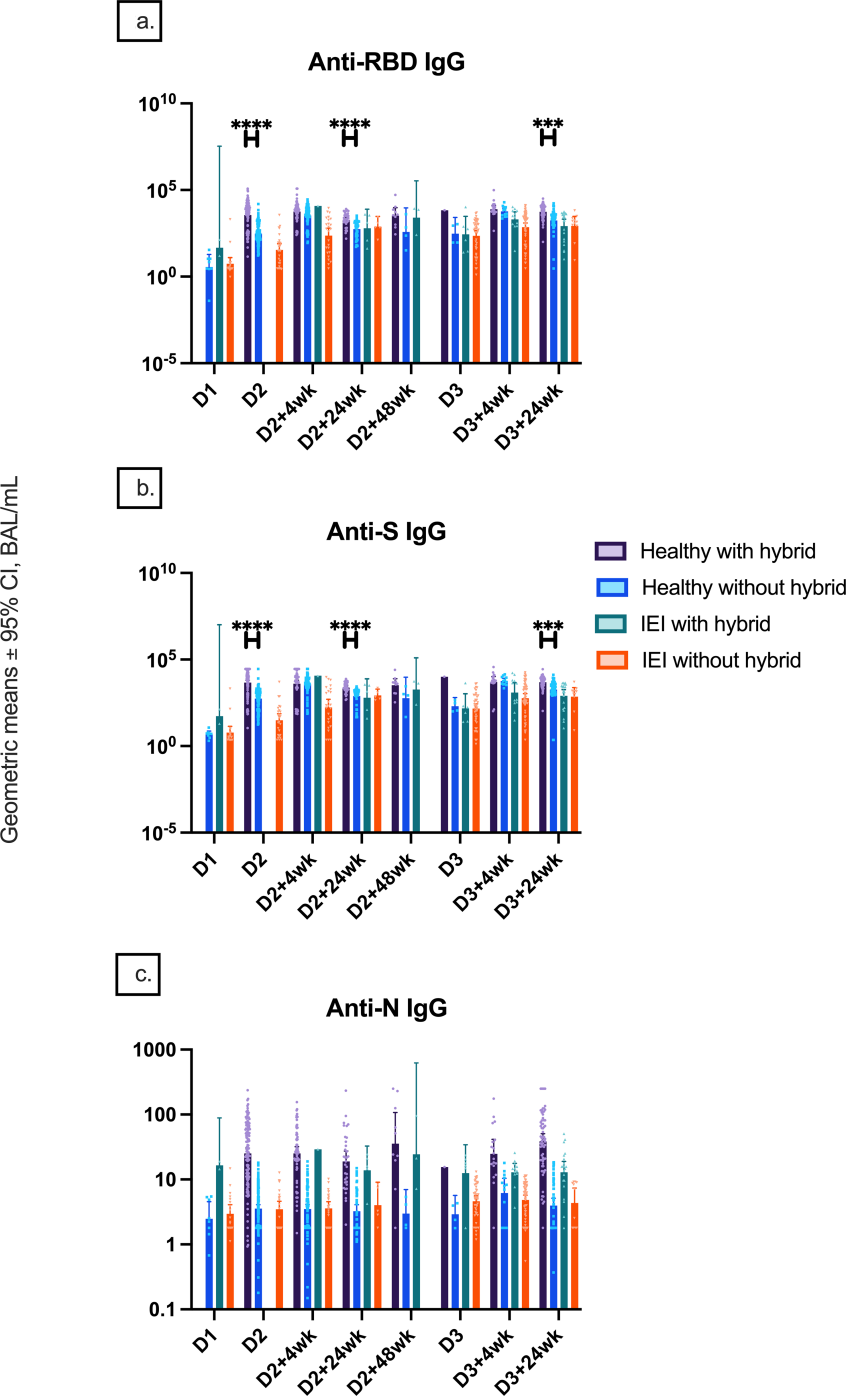
Natural and hybrid immunity. Geometric mean ± 95% confidence interval of **(A)** anti-RBD IgG responses by hybrid immunity status and group at different timepoints pre- and post-COVID-19 vaccination up to 24 weeks after dose 3, **(B)** anti-S IgG responses by hybrid immunity status and group at these different timepoints, and **(C)** anti-N IgG serology by hybrid immunity status and group at these different timepoints. *** indicates p < 0.001, **** indicates p < 0.0001, and D1, before dose 1; D2, before dose 2; D2 + 4wk, 4 weeks after dose 2; D2 + 24wk, 24 weeks after dose 2; D2 + 48wk, 48 weeks after dose 2; D3, before dose 3; D3 + 4wk, 4 weeks after dose 3; D3 + 24wk, 24 weeks after dose 3; anti-RBD, anti-receptor binding domain; anti-S, anti-spike protein; anti-N, anti-nucleocapsid; IEIs, inborn errors of immunity; CI, confidence interval.

**Figure 3:**
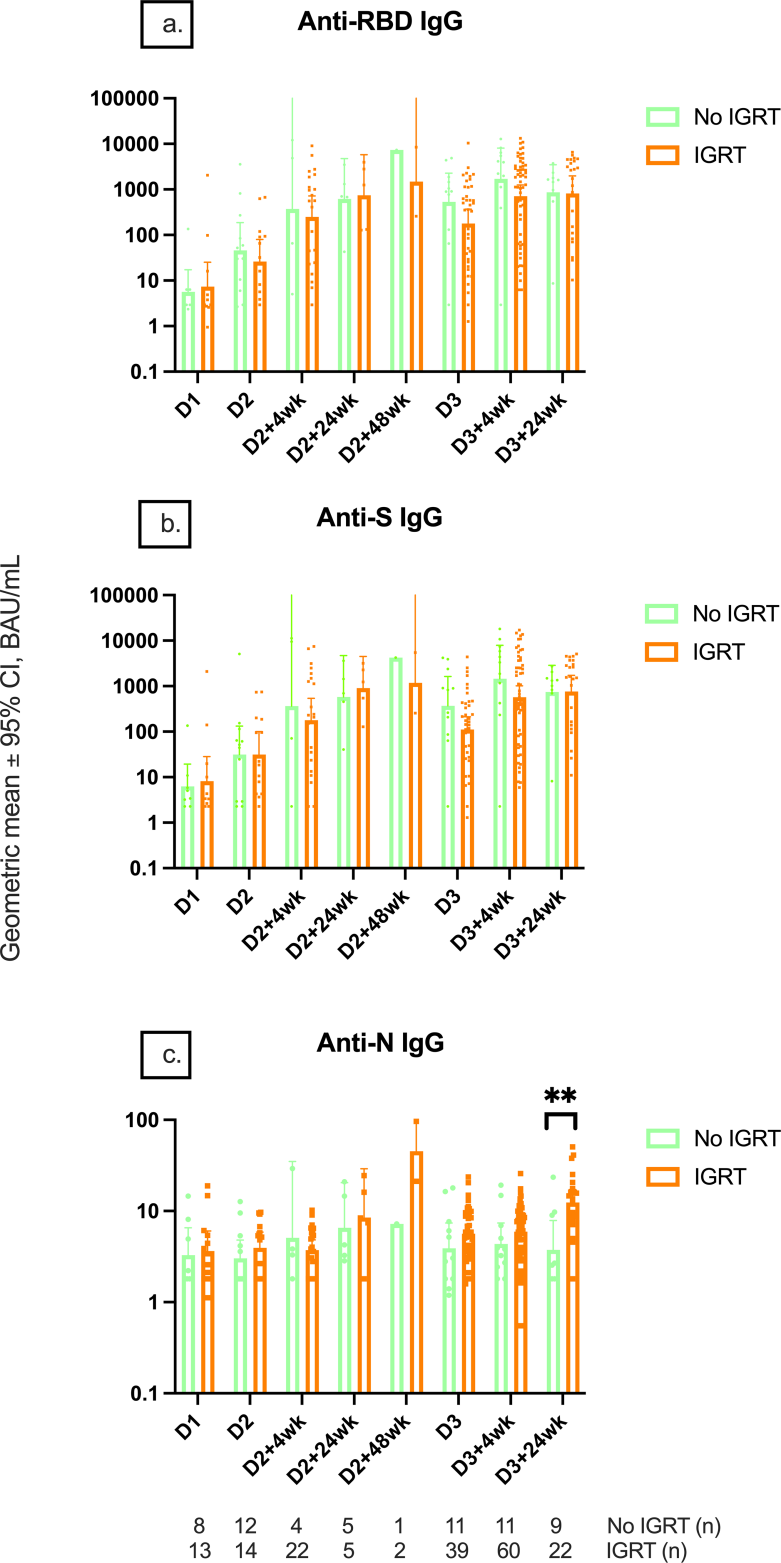
Serological responses among IEI participants receiving IGRT or not receiving IGRT. Geometric mean IgG serology of **(A)** anti-RBD, **(B)** anti-S, and **(C)** anti-N IgG titers between IEI participants receiving and not receiving immunoglobulin replacement therapy at different timepoints pre- and post-COVID-19 vaccination up to 24 weeks after dose 3. Number (*n*) immunodeficient participants with data included per timepoint are indicated. ** indicates p < 0.01. Error bars indicate the 95% CI. D1, before dose 1; D2, before dose 2; D2 + 4wk, 4 weeks after dose 2; D2 + 24wk, 24 weeks after dose 2; D2 + 48wk, 48 weeks after dose 2; D3, before dose 3; D3 + 4wk, 4 weeks after dose 3; D3 + 24wk, 24 weeks after dose 3; IGRT, immunoglobulin replacement therapy; CI, confidence interval; anti-RBD, anti-receptor binding domain; anti-S, anti-spike protein; anti-N, anti-nucleocapsid; IEI, inborn error of immunity.

SARS-CoV-2 infections before vaccine doses 2 and 3 and within 6 months of receiving dose 3 were reported in 15/37 (40.5%), 5/37 (13.5%), and 11/37 (29.7%) of controls and 1/149 (0.7%), 9/149 (6.0%), and 32/149 (21.5%) of IEIs. Anti-RBD and anti-S IgG titers were only higher in controls with hybrid immunity compared to controls without hybrid immunity at D2, D2 + 24wk, and D3 + 24wk, while no difference was found in IEIs with or without hybrid immunity at any timepoint (**Figures 2A, B**). Anti-N IgG titers of controls increased at an earlier timepoint, whereas the titers increased at a later timepoint in IEI participants, consistent with the report of SARS-CoV-2 infection (**Figure 2C**). Further, anti-N IgG titers were higher in both healthy and IEI participants with hybrid immunity than those without hybrid immunity (**Figure 2C**). Interestingly, the higher anti-N IgG titers at D3 + 24wk in IEIs were driven by IEI participants on IGRT (12.9 [9.1–18.28] vs. 3.76 [1.79–7.89], p = 0.0008) (**Figure 3**).

### Impact of IGRT on serological responses

To evaluate if measured serological responses in IEIs were impacted by IGRT, we analyzed serology data based on IGRT (**Figure 3**). IGRT did not impact anti-RBD and anti-S IgG titers at any timepoint. This was confirmed by the analysis of anti-S IgG levels in IEI participants in the PAD subgroup (i.e., with predominant B-cell deficiency), as they were the most likely to have the poorest HI response to the vaccine (**Supplementary Material**). In fact, PAD participants who were on IGRT tended to have lower anti-S IgG (not statistically significant) and anti-S IgA than those not on IGRT.

### Neutralization antibody response

**Figure 4:**
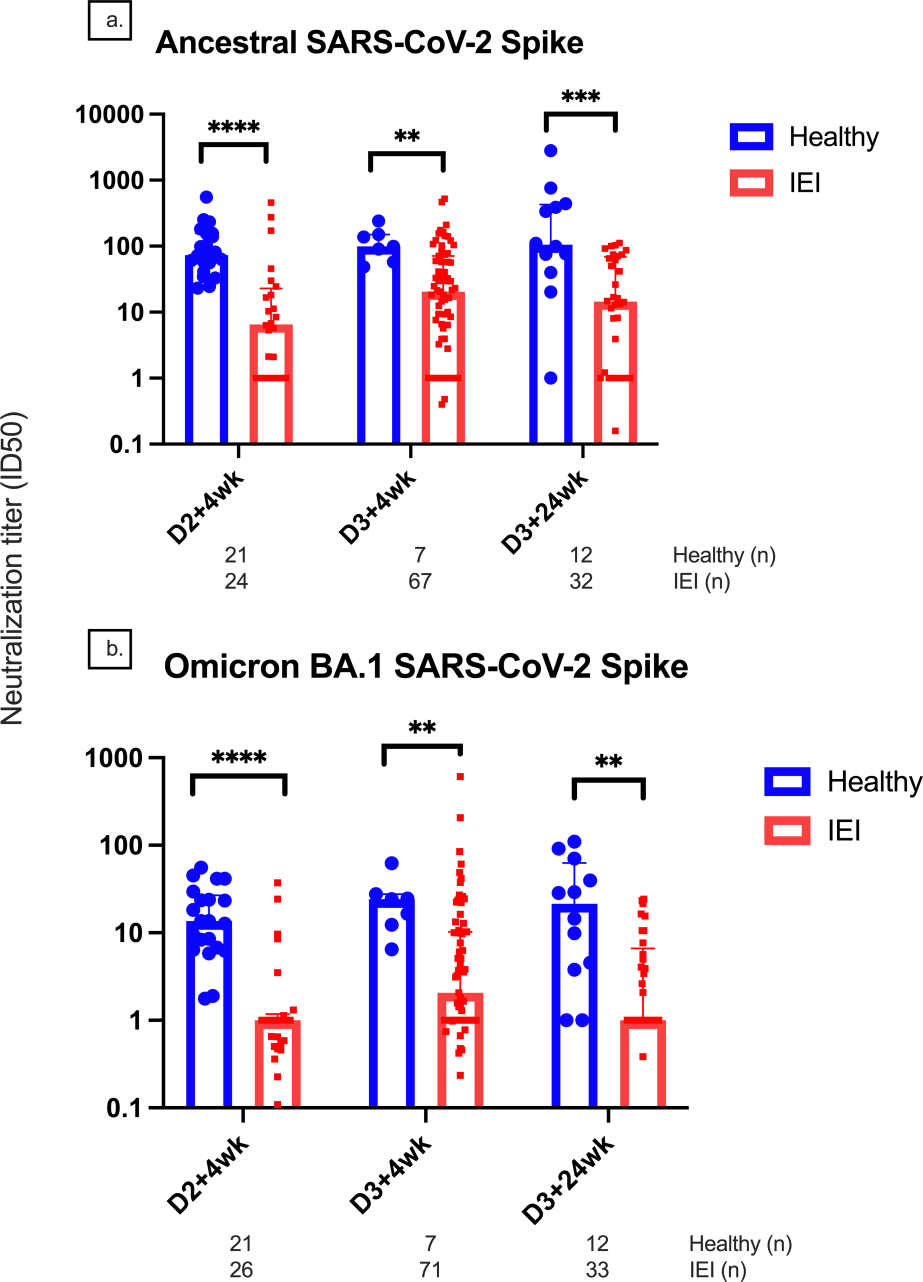
Neutralization titers for the ancestral and Omicron BA.1 variants. Comparison of neutralization titers at 4 weeks after doses 2 and 3 between healthy and immunodeficient participants for the **(A)** ancestral and **(B)** Omicron BA.1 variants. Number (*n*) of healthy and IEI participant data included per timepoint are indicated. ** indicates p < 0.01, *** indicates p < 0.001, **** indicates p < 0.0001. The error bars indicate the standard deviation. D1, before dose 1; D2, before dose 2; D2 + 4wk, 4 weeks after dose 2; D2 + 24wk, 24 weeks after dose 2; D2 + 48wk, 48 weeks after dose 2; D3, before dose 3; D3 + 4wk, 4 weeks after dose 3; D3 + 24wk, 24 weeks after dose 3; IGRT, immunoglobulin replacement therapy; CI, confidence interval; IQR, interquartile range; anti-RBD, anti-receptor binding domain; anti-S, anti-spike protein; anti-N, anti-nucleocapsid.

At D2 + 4wk, D3 + 4wk, and D3 + 24wk, the median (IQR) ID50 against the ancestral strain was significantly lower in IEIs compared to controls (6.55 [1.00, 22.9] vs. 73.4 [48.6, 155.8], p < 0.0001; 20.34 [3.9, 71] vs. 99.4 [57.5, 150.6], p = 0.0032; 14.7 [1, 68.9] vs. 104.9 [48.6, 426.6], p = 0.0005, respectively) (**Figure 4**). Similar results were seen against Omicron BA.1 variant, where IEIs had a significantly lower ID50 compared to controls at D2 + 4wk, D3 + 4wk, and D3 + 24wk (1 [0.5, 1.8] vs. 13.6 [6.6, 27.7], p < 0.0001; 2.1 [1, 10.3] vs. 24.3 [12.3, 27.6], p = 0.0021; 1 [1, 6.6] vs. 21.5 [4, 62.7], p = 0.0013, respectively). There were reduced ID50 titers against the Omicron BA.1 variant in both groups as compared to those against the ancestral strain.

### Cell-mediated vaccine response

**Figure 5:**
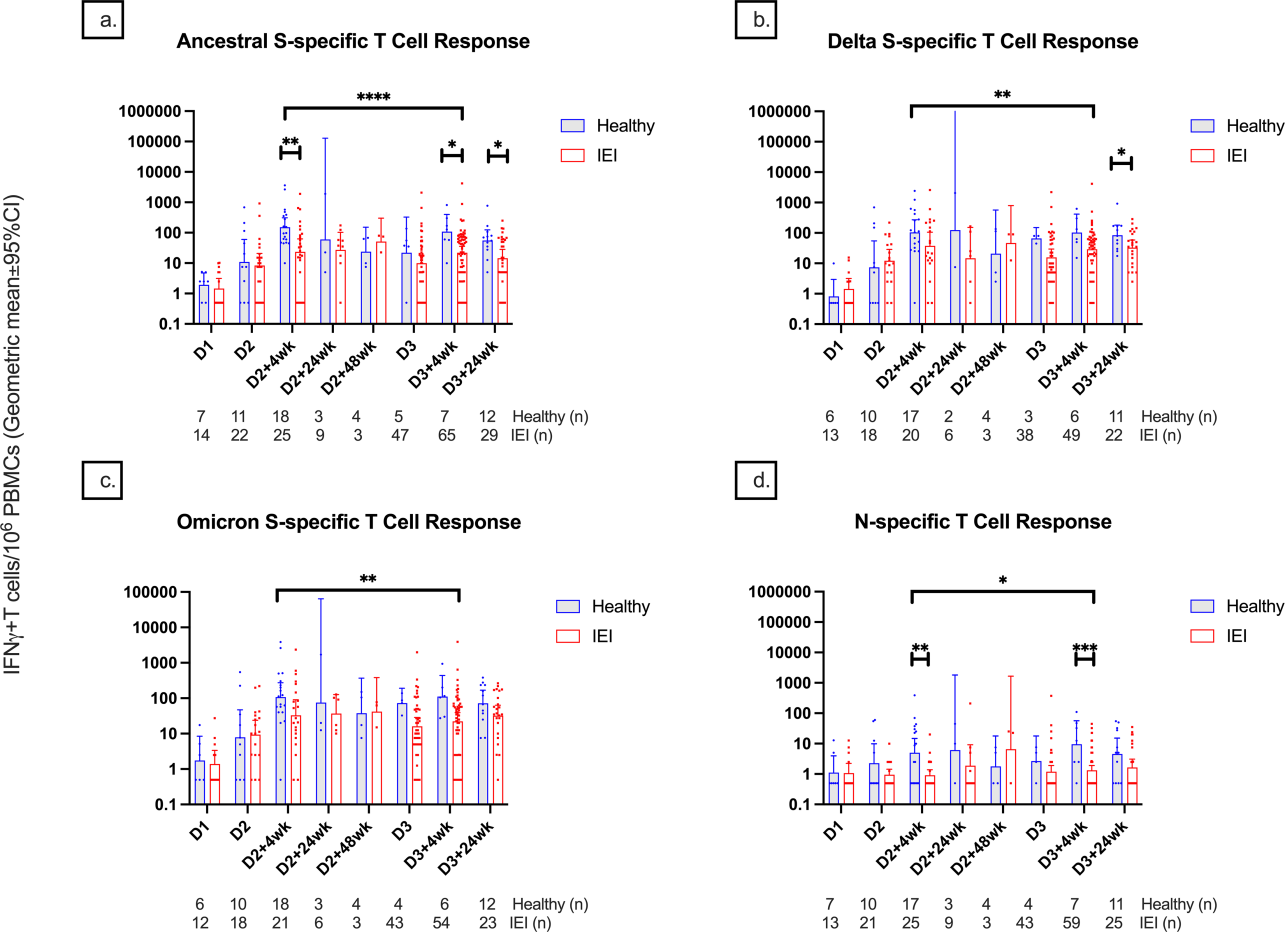
T-cell responses among healthy controls and participants with inborn errors of immunity at different timepoints pre- and post-COVID-19 vaccination up until 24 weeks after dose 3. T-cell responses against the **(A)** spike protein of the ancestral variant, **(B)** spike protein of the delta variant, **(C)** spike protein of the B.1.1.529 Omicron variant, and **(D)** nucleocapsid protein are depicted and measured as IFN-γ positive T cells per 1 million PBMCs. Number (*n*) of healthy and immunodeficient participant data included per timepoint are indicated. * indicates p < 0.05, ** indicates p < 0.01, *** indicates p < 0.001, **** indicates p < 0.0001. The error bars indicate the 95% confidence interval. S, spike; N, nucleocapsid; IFN-γ, interferon gamma cytokine; PBMCs, peripheral blood mononuclear cells; D1, before dose 1; D2, before dose 2; D2 + 4wk, 4 weeks after dose 2; D2 + 24wk, 24 weeks after dose 2; D2 + 48wk, 48 weeks after dose 2; D3, before dose 3; D3 + 4wk, 4 weeks after dose 3; D3 + 24wk, 24 weeks after dose 3.

The T-cell responses measured by S-specific IFN-γ^+^ T cells/10^6^ PBMCs at D2 + 4wk, D3 + 4wk, and D3 + 24wk were significantly lower in IEIs compared to controls (23.6 [95% CI, 8.8–63.1] vs. 163.0 [77.9–340.9], p = 0.002; 21.65 [13.50–34.70] vs. 109.00 [29.85–398.02], p = 0.03; 13.7 [6.8–27.4] vs. 55.9 [25.0–124.6], p = 0.02) (**Figure 5A**).

There was no significant difference in S-specific T-cell responses against S-Delta and B.1.1.529-S-Omicron responses between IEIs and controls at any timepoint except at D3 + 24wk, where there was a significantly lower response in IEIs compared to controls (31.5 [16.8–57.5] vs. 84.3 [39.3–180.8], p = 0.048) (**Figures 5B, C**).

IEIs had lower T-cell response than controls at 4 weeks but not at 6 months after completing their respective primary series (p < 0.0001 for S-ancestral, p = 0.007 for S-Delta, and p = 0.03 for B.1.1.529-S-Omicron). Similar to the serological response, T-cell response to natural infection was more evident in controls than in IEI at D2 + 4wk and D3 + 4wk (**Figure 5D**), corresponding with their history of prior SARS-CoV-2 infections.

### Responses by IEI subgroups

**Figure 6:**
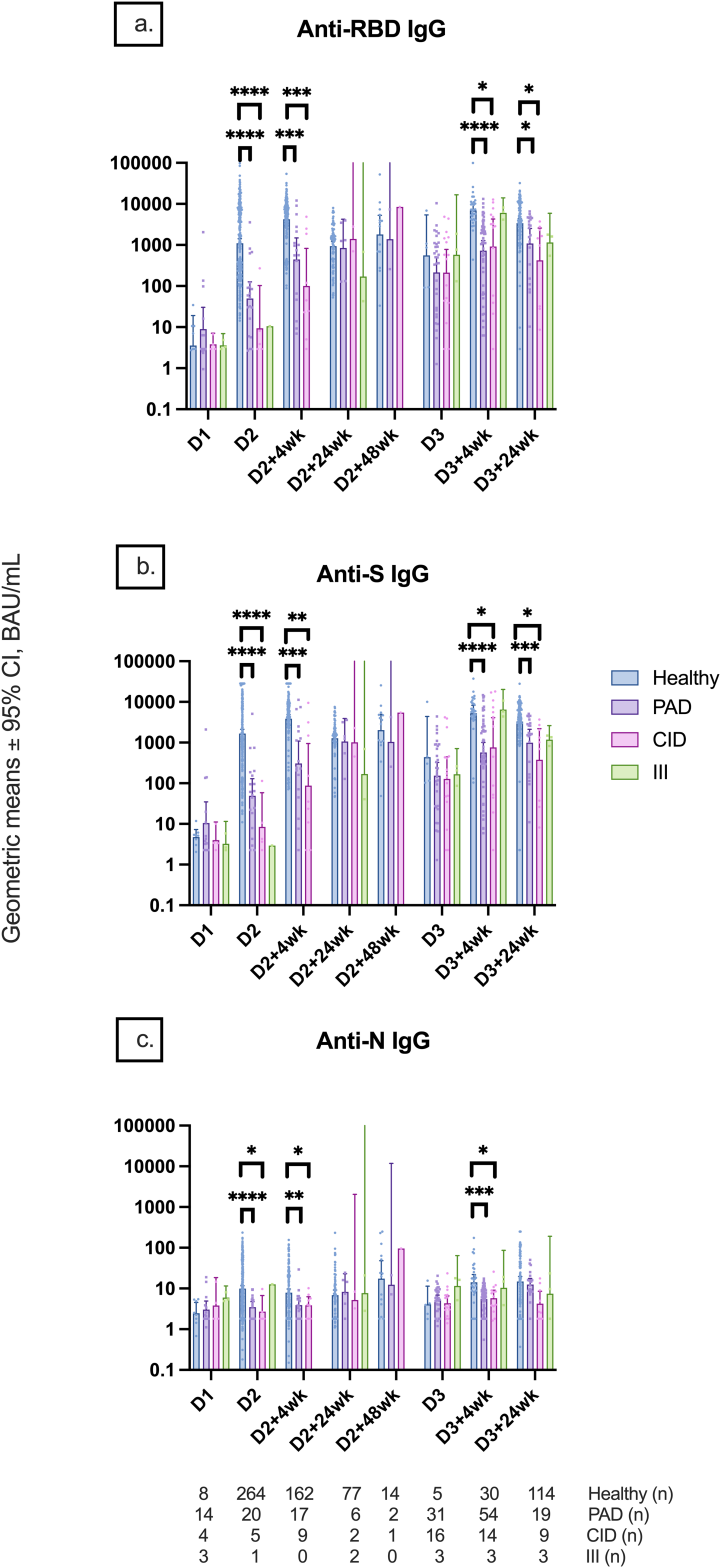
Geometric mean IgG serology levels by participant subgroups. Comparison of **(A)** anti-RBD IgG, **(B)** anti-S IgG, and **(C)** anti-N IgG titers among participant subgroups (healthy, PAD subgroup, CID subgroup, and III subgroup) at different timepoints pre- and post-COVID-19 vaccination up to 24 weeks after dose 3. Statistical analysis was not performed for the III subgroup due to the small sample size. Number (*n*) of healthy and immunodeficient subgroup participants with data included per timepoint are indicated. * indicates p < 0.05, ** indicates p < 0.01, *** indicates p < 0.001, **** indicates p < 0.0001. Error bars indicate the 95% CI. D1, before dose 1; D2, before dose 2; D2 + 4wk, 4 weeks after dose 2; D2 + 24wk, 24 weeks after dose 2; D2 + 48wk, 48 weeks after dose 2; D3, before dose 3; D3 + 4wk, 4 weeks after dose 3; D3 + 24wk, 24 weeks after dose 3; CI, confidence interval; PAD, primary antibody deficiency; CID, combined immunodeficiency; III, intrinsic innate immune defect.

**Figure 7:**
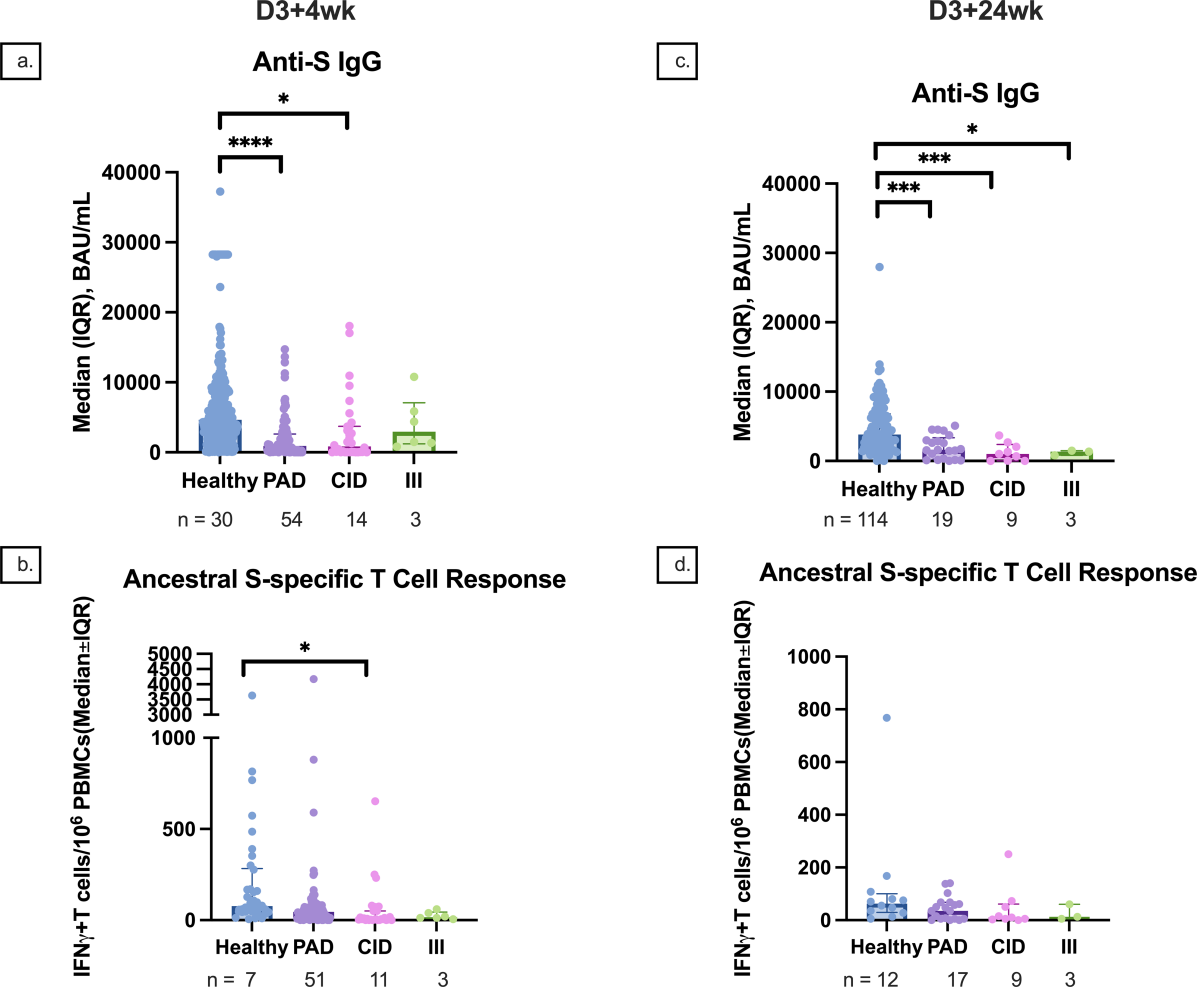
Median anti-S IgG and T-cell responses by participant subgroup at 4 and 24 weeks after dose 3. **(A)** Median anti-S IgG responses at 4 weeks after dose 3 compared between healthy, PAD, CID, and III subgroup participants. **(B)** Median S-specific T-cell responses against the ancestral strain at 4 weeks after dose 3 compared between healthy, PAD, CID, and III subgroup participants. **(C)** Median anti-S IgG responses at 24 weeks after dose 3 compared between healthy, PAD, CID, and III subgroup participants. **(D)** Median S-specific T-cell responses against the ancestral strain at 24 weeks after dose 3 compared between healthy, PAD, CID, and III subgroup participants. * indicates p < 0.05, *** indicates p < 0.001, **** indicates p < 0.0001. Error bars indicate the 95% CI. D3 + 4wk, 4 weeks after dose 3; D3 + 24wk, 24 weeks after dose 3; IQR, interquartile range; S, spike protein; PAD, primary antibody deficiency; CID, combined immunodeficiency; III, intrinsic innate immune defect.

Participants in the PAD subgroup mounted weaker anti-RBD, anti-S, and anti-N responses at D2, D2 + 4wk, D3 + 4wk, and D3 + 24wk compared to controls (**Figure 6**). Similar differences were also seen in the CID subgroup (**Figure 6**). Although there was no statistically significant difference in anti-S IgG between the PAD and CID subgroups, the PAD subgroup had lower median anti-S IgG than the CID and III subgroups at D3–4 (533.1 [295.5, 2,512.0] vs. 3,105.7 [52.0, 7,338.8] vs. 5,866.3 [4,377.1, 10,777.2], respectively) (**Figures 6**, **7**). Conversely, S-specific T-cell response was higher although not statistically significant in the PAD subgroup than in the CID subgroup at D3 + 4wk (46.3 [12.5,85] vs. 5 [2.5, 50], p = 0.07). HI comparisons were performed; however, CMI comparisons with the III subgroup were not performed due to a small sample size (**Figure 7**).

### Correlation between HI and CMI

**Figure 8:**
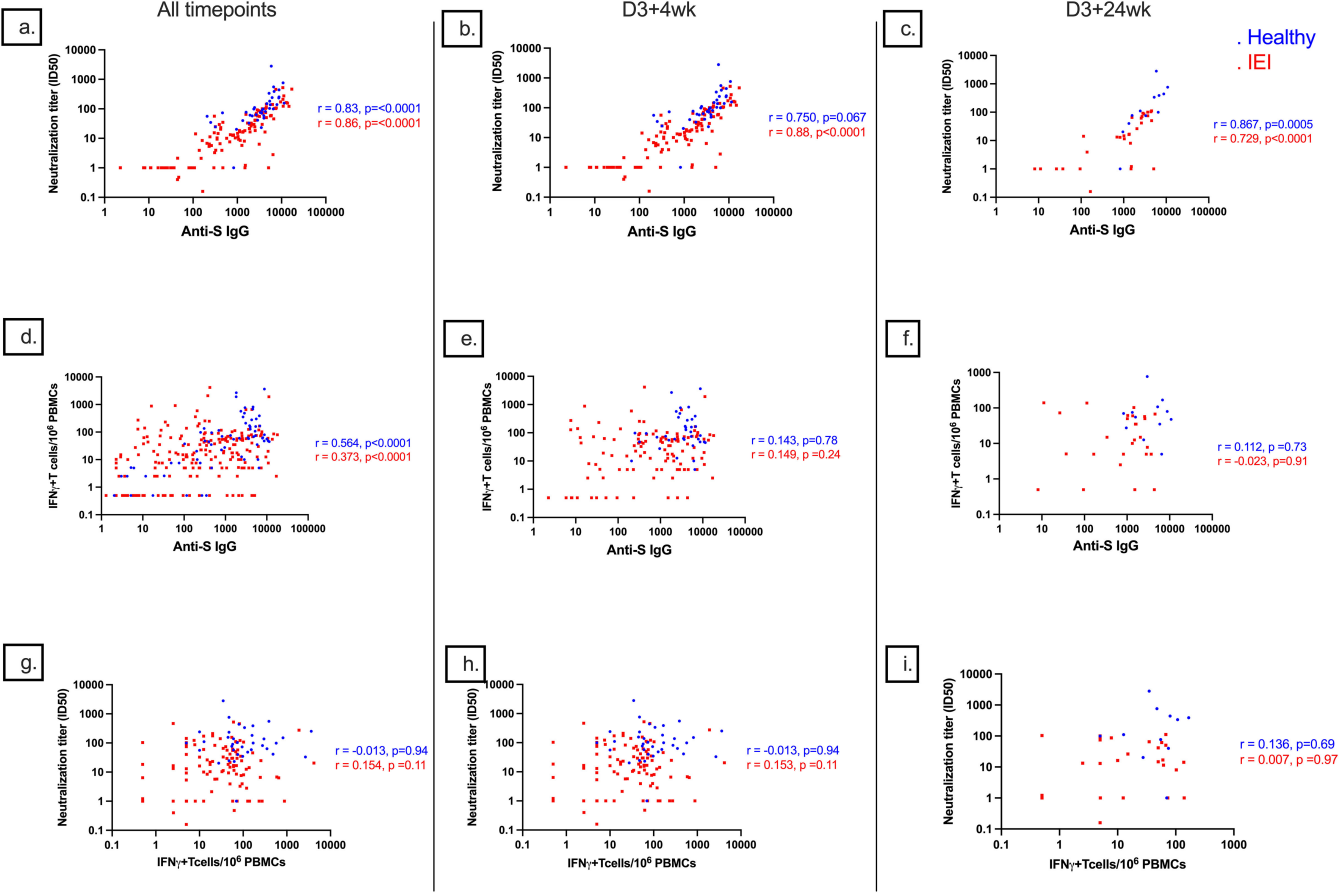
Correlations of anti-S IgG, ID50, and S-specific T-cell responses. Correlations of **(A)** anti-S IgG and ID50 with combined data from all timepoints, **(B)** anti-S IgG and ID50 at D3 + 4wk, **(C)** anti-S IgG and ID50 at D3 + 24wk, **(D)** anti-S IgG and S-specific T-cell responses with combined data from all timepoints, **(E)** anti-S IgG and S-specific T-cell responses at D3 + 4wk, **(F)** anti-S IgG and S-specific T-cell responses at D3 + 24wk, **(G)** S-specific T-cell responses and ID50 with combined data from all timepoints, **(H)** S-specific T-cell responses and ID50 at D3 + 4wk, and **(I)** S-specific T-cell responses and ID50 at D3 + 24wk. D3 + 4wk, 4 weeks after dose 3; D3 + 24wk, 24 weeks after dose 3; S, spike; IQR, interquartile range; S, spike; IFN-γ, interferon gamma; PBMC, peripheral blood mononuclear cell.

The degree of correlation of markers of HI and CMI against S-protein differed between healthy and IEI participants (**Supplementary Material**, **Figure 8**). In general, there was a strong correlation between anti-S IgG and ID50 (r = 0.83, p < 0.0001 for controls and 0.86, p < 0.0001 for IEI participants), a moderate correlation between serologies and cellular responses (r = 0.564, p < 0.0001 for healthy and 0.373, p < 0.0001 for IEI), but no correlation between ID50 and cellular responses (r = −0.013, p = 0.94 for controls and 0.154, p = 0.11 for IEI participants). At specific timepoints (**Figure 8**), there was also a strong correlation between anti-S IgG and ID50 at D3 + 4wk (r = 0.750, p = 0.067 for controls and 0.88, p < 0.0001 for IEI participants) and D3 + 24wk (r = 0.867, p = 0.0005 for controls and 0.729, p < 0.0001 for IEI participants). There was weak to no correlation between serologies and cellular responses at D3 + 4wk (r = 0.143, p = 0.78 in controls and 0.149, p = 0.24 in IEI participants) and at D3 + 24wk (r = 0.112, p = 0.73 in controls and −0.023, p = 0.91 in IEI participants). Similarly, there was weak to no correlation between ID50 and cellular responses at D3 + 4wk (r = −0.013, p = 0.94 in controls and 0.153, p = 0.11 in IEI participants) and at D3 + 24wk (r = 0.136, p = 0.69 in controls and 0.007, p = 0.97 in IEI participants).

### Vaccine safety

Among participants enrolled between D1 and D3 + 24wk, there were 21 MAAEs in 17 (11.4%) IEI participants and only one MAAE in controls (**Table 2**). For IEI participants, over 90% of these events were considered moderate severity. Of all MAAEs, 80.95% were determined to be unrelated to vaccination, while the remaining events were identified as probably (4.8%) or possibly (9.5%) related to vaccination. The average onset of MAAEs in IEI participants was 88.3 days from a vaccination, all of which occurred between D3 and D3 + 24wk. The MAAE in the controls was a spontaneous abortion reported 2 weeks after the first vaccination at approximately 7 weeks of gestation and determined to be possibly related to vaccination.

**Table 2:** Medically attended COVID-19 infection events between D1 and D3–24 during the study.

	Healthy (n = 37)	IEI (n = 149)
Number of events, n (%)	1	13
Number of patients, n (%)	1 (2.7)	13 (8.7)
Severity, n (%)		
Mild—no medical intervention	0	0
Moderate—minimal intervention (medication, medically attended)	1 (100.0)	13 (100)
Severe—hospitalization	0	0
Serious—life threatening	0	0
Death—fatal	0	0
Infection events by period		
Mar 2020–Dec 2020	0	0
Jan 2021–May 2021	0	0
Jun 2021–Dec 2021	0	1 (7.7)
Jan 2022–Mar 2022	0	8 (61.5)
Apr 2022–Jun 2022	1 (100.0)	4 (30.8)
Jul 2022–Oct 2023	0	0
Infection events by vaccination		
Before D1	0	0
Between D1 and D2	0	0
Between D2 and D3	1 (100.0)	0
Between D3 and D3 + 24wk	0	13 (100)
Treatment received		
Nirmatrelvir/ritonavir	0	2 (15.4)
Remdesivir	0	1 (7.7)
Sotrovimab	0	6 (46.2)
Tocilizumab	0	0
Dexamethasone	0	0
No treatment ^a^	1 (100.0)	5 (38.5)

IEI, inborn error of immunity.

a No treatment of antivirals or mABs listed in this table was given for the reported infections; however, other types of treatments may have been used but not recorded.

### SARS-CoV-2 infection

Overall, including infection history from before and after study enrolment, we found that 24 (64.9%) of controls reported at least one SARS-CoV-2 infection with a total of 31 infections, and 15/31 occurred when the participant was vaccine-naive. However, 40 (26.9%) of IEI participants had at least one SARS-CoV-2 infection. The total number of reported SARS-CoV-2 infections was 42, and only one occurred before any vaccination (**Supplementary Material**). Nine (21.43%) infections were reported between doses 2 and 3 for IEIs, while 5 (16.13%) were reported for healthy controls. Most infections reported by IEI participants occurred after dose 3 (32, 76.19%) and after January 2022 (35, 82.33%).

During the study, 8.7% of IEI participants and 2.7% of controls had medically attended COVID-19 infections (p = 0.31) (**Table 2**). No severe infections were reported during the study, but there was one severe infection requiring 8 days of hospitalization reported from one IEI patient prior to study enrolment.

**Figure 9:**
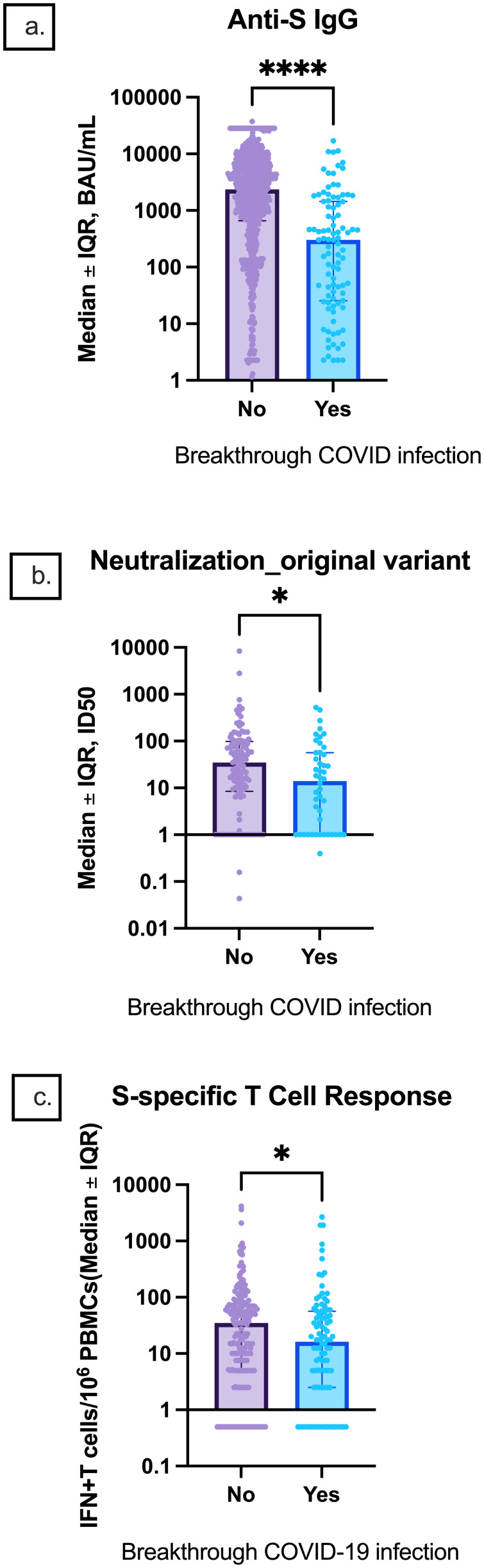
Comparison of pre-infection median anti-S IgG, neutralization titers, and S-specific T-cell responses between participants with and without breakthrough infection. Median **(A)** anti-S IgG, **(B)** original strain ID50, and **(C)** S-specific T-cell responses are depicted. Error bars indicate the IQR. IQR, interquartile range; S, spike; IFN-γ, interferon gamma; PBMC, peripheral blood mononuclear cell. * indicates p < 0.05, **** p < 0.0001.

Participants with breakthrough infections had lower median anti-S IgG titers, ID50 titers, and S-specific T-cell responses prior to infection than participants who did not have breakthrough infections [301.7 (25.5, 1,445.0) vs. 2,367 (656.7, 6,507.0) BAU/mL, p < 0.0001; 14.0 (1, 56.6) vs. 34.7 (8.5, 98.3), p = 0.0.4; 16.25 (2.5, 56.6) vs. 35 (5.0, 75.0), p = 0.03, respectively] (**Figure 9**).

## Discussion

In this study, we compared HI and CMI responses to COVID-19 vaccines in participants with IEIs and controls. Consistent with previous studies, HI responses at D2 + 4wk and D3 + 4wk were significantly lower in participants with IEIs compared to controls (30, 31). Nevertheless, antibodies produced by IEIs were functional. CMI responses in IEI patients remain relatively understudied compared to HI. We observed that IEI patients could mount T-cell response to spike, although lower than that of controls after doses 2 and 3 (32). Reassuringly, both antibody and T-cell responses were durable and remained at comparable levels at 6 months after dose 3 to those at 4 weeks after dose 3 in IEI patients, whereas these responses waned moderately over time in controls (**Supplementary Material**). Similarly, anti-S IgG responses were examined in a study by Zendt et al. and were also found to persist from 4 weeks to 6 months after dose 3 (30). In our study, given that the response waned over time in controls but persisted in IEIs, this resulted in no significant difference in serological responses between the two groups at 24 weeks after their respective primary series.

Due to rapid changes in circulating viral strains, we also examined the neutralizing antibody titer against the BA.1 Omicron variant and T-cell response against B.1.1.529-Omicron-spike. We found that antibodies generated by ancestral COVID-19 vaccines had reduced neutralizing capacity to the Omicron variant compared to the ancestral strain, which is consistent with other studies (30, 33). For instance, Zendt et al. found that their participants with immunodeficiency had significantly lower anti-S IgG and as well as similar but lower ACE2 inhibition against the Omicron BA.1 variant compared to other variants (30). However, we did observe a relatively stable T-cell response to B.1.1.529-S-Omicron. In addition to IgG response, IgA and IgM responses to vaccines were also lower in IEI participants than in controls. Anti-S IgG correlated very well with neutralizing antibody titer, particularly in IEI patients, and there was a moderate correlation between anti-S IgG and T-cell response in both groups. However, T-cell response did not correlate well with neutralizing antibody titers.

Despite severe COVID-19 infections being a concern in the IEI community, our study reported only one severe COVID-19 infection that occurred before the completion of the primary series. Durable T-cell response may have protected IEI patients from severe outcomes (34–37). As expected, we found that low anti-S IgG titers, ID50, and S-specific T cells were associated with breakthrough infections. Most SARS-CoV-2 infections occurred after December 2021 when the Omicron variant dominated. This temporal increase in post-vaccination infection was also noted in the Chen et al. real-world assessment of immunogenicity in immunocompromised individuals following SARS-CoV-2 vaccination, where they also attributed the increase in cases at this time to the emergence of the Omicron variant (33).

Many studies have reported that participants with hybrid immunity (i.e., a history of SARS-CoV-2 vaccination and prior SARS-CoV-2 infection) mount stronger HI responses compared to vaccine-induced immunity (without prior history of infection) or natural immunity alone (38–41). These findings are consistent with our study of controls; however, we did not observe this pattern in IEI participants. There was no difference in HI responses in IEI participants with hybrid immunity compared to IEI participants without hybrid immunity at any timepoint. This evidence suggests that the presence of impaired immune response in IEIs was not only to vaccination but also to infection. We also speculated that this lack of difference in HI responses between IEIs with and without hybrid immunity could be due to IGRT. However, we found that IG products started to contain anti-S IgG only in late 2021 or early 2022 based on our X-linked agammaglobulinemia (XLA) patients’ data who should not have any antibody response to vaccines (42–44) yet had very high anti-S IgG in late 2021 and early 2022 (**Supplementary Material**). It is known that manufacturing IG products from plasma donation can take up to 1.5 years (45, 46). As such, it is very likely that IG products administered to patients in our study did not contain anti-S IgG until 1–2 years after the start of COVID-19 vaccine campaigns. In addition, we did not observe a difference between anti-S IgG in IEI participants on IGRT compared to IEI participants not on IGRT. This is also consistent with other studies where there was no significant difference in anti-S IgG concentrations between patients on IGRT and those not on IGRT at all timepoints (30). Thus, IGRT did not explain the lack of difference in HI responses between IEIs with and without hybrid immunity.

Participants with combined B- and T-cell deficiency had lower CMI response compared with participants with B-cell deficiencies, and there was no significant difference between CMI response in healthy and B cell-deficient participants. Importantly, we observed two patients with predominant B-cell deficiency who had S-specific T-cell responses that were higher than those in controls (**Figure 5**). This suggests that B cells are not crucial in mounting a functional T-cell response to COVID-19 vaccine. Indeed, this was further supported in a study conducted by Guiterrez-Bautista et al., where they found that all 26 CVID patients and three of four XLA patients mounted a positive cellular response to doses 2 and 3 (31).

The anti-N antibody response at D3 + 24wk increased in IEIs when compared to D3 + 4wk, while the response decreased in controls. This is consistent with the timing of the reported SARS-CoV-2 infections in the study. Additionally, it is established that participants with IEIs can have a higher frequency of viral persistence and prolonged COVID-19 compared to controls and may in part explain the durability of anti-S IgG, IgA, and IgM at D3 + 24wk (47, 48).

The large sample size of 149 IEI cases is a major strength of this study, as this is the first collaboration of its size across Canada to assess post-vaccination responses in individuals with IEIs. Additionally, this is one of the few studies to examine CMI responses to COVID-19 vaccination in IEIs, particularly IEIs other than PAD. Nonetheless, this study has several limitations. First, due to limited control recruitment, additional serological data were collected from the SSO study, as described. Therefore, variables such as recruitment strategy and technical and methodological differences may vary between our cohort and SSO controls. An assessment of vaccine safety and effectiveness was also limited with a small number of controls. Further, this study only examined HI and CMI responses up to D3 + 24wk. In Canada, individuals with IEIs are recommended to receive COVID-19 vaccination every 6 months with the updated vaccines. Therefore, more research on the immune responses to additional doses is being conducted by our group. Finally, subgroup analysis of vaccine response particularly in IEI participants with innate immune defects was limited by the small sample size.

To conclude, IEI patients can elicit both HI and CMI responses that are durable at least 6 months after their primary series, but the degrees of responses vary depending on underlying immune defects and generally are lower than those of healthy individuals. Although the correlation between S-specific antibodies and viral neutralizing titers is strong, the correlation between HI and CMI is not similarly seen. We did not observe a concerning vaccine safety signal. This study supports the Canadian vaccine recommendations at the time that the study was conducted, which recommended a three-dose primary series in people with IEIs.

## Data Availability

The original contributions presented in the study are included in the article/**Supplementary Material**. Further inquiries can be directed to the corresponding author.
