# Supplementary material for: Humoral and cell-mediated immune responses to COVID-19 vaccines up to 6 months post three-dose primary series in adults with inborn errors of immunity and their breakthrough infections
Source: Front Immunol. 2025 Jan 21;15:1501908. doi: 10.3389/fimmu.2024.1501908 (PMC11790575; doi:10.3389/fimmu.2024.1501908)

# SUPPLEMENTAL FILE

**Appendix A** List of VISID co-investigators

**Table S1.**  History of COVID-19 vaccination prior to study enrolment

**Table S2.** Known molecular diagnoses of participants with IEI

**Table S3.** Geometric mean ratio of serological responses

**Table S4.**  COVID-19 infection events recorded prior and during the study

**Table S5.** Medically attended adverse events (non-SARS-CoV-2 events)

**Figure S1.** Study scheme

**Figure S2.** Summary of participant recruitment and number of participant visits at each timepoint

**Figure S3.**  Geometric mean anti-S IgG responses by participant sex

**Figure S4.** Baseline IgA and geometric mean anti-S IgA serology in participants with and without low baseline IgA

**Figure S5.** Presence of anti-S IgG in participant with X-linked agammaglobulinemia

**Figure S6.** Anti-N immunoglobulin titers among IEI participants receiving and not receiving IGRT

**Figure S7.** Anti-S IgG and anti-S IgA among PAD participants receiving and not receiving IGRT

**Figure S8.** Correlation matrices of serological and cellular responses after Doses 2 and 3

**1. APPENDIX**

**List of co-investigators of the VISID Study Group**

**Principal Investigator:**

Dr. Juthaporn Cowan, University of Ottawa, ON

**Co-Principal Investigators:**

Dr. Donald Vinh, McGill University, QC

Dr. Emilia Falcone, IRCM, Montreal, QC

Dr. Hugo Chapdelaine, IRCM, Montreal, QC

**Co-Investigators:**

Dr. Karina Top, Dalhousie University, NS

Dr. Beata Derfalvi, Dalhousie University, NS

Dr. Alejandro Palma, Dalhousie University, NS

Dr. Thomas Issekutz, Dalhousie University, NS

Dr. Lisa Barrett, Dalhousie University, NS

Dr. Sharon Oldford, Dalhousie University, NS

Dr. Gina Lacuesta, Dalhousie University, NS

Dr. Hélène Decaluwe, CHU Ste Justine, QC

Dr. Dana Unninayar, University of Ottawa, ON

Dr. Marc-Andre Langlois, University of Ottawa, ON

Dr. Anne Pham-Huy, CHEO, Ottawa, ON

Dr. Julia Upton, University of Toronto, ON

Dr. Stephen Betschel, Unity Health Toronto, ON

Dr. Tamar Rubin, University of Manitoba, MB

Dr. Sneha Suresh, University of Alberta, AB

Dr. Nicola Wright, University of Calgary, AB

Dr. Luis Murguía-Favela, University of Calgary, AB

Dr. Tatiana Kalashnikova, University of Calgary, AB

Dr. Manish Sadarangani, BC Children’s Hospital, BC

Dr. Arianne Buchan, The Ottawa Hospital Research Institute, ON

Dr. Tim Ramsay, The Ottawa Hospital Research Institute, ON

Dr. Elie Haddad, CHU Ste Justine, QC

Dr. Mohammed Osman, University of Alberta, AB

Dr. Bruce Ritchie, University of Alberta, AB

Dr. Kyla Hildebrand, University of British Columbia, BC

Dr. Fabien Touzot, CHU Ste Justine, QC

**2. TABLES**

**Table S1**. History of COVID-19 vaccination prior to study enrolment

|  | Healthy  (N = 37) | SOS Healthy cohort  (N = 386) | Inborn Error of Immunity^a^  (N = 149) | | | | |
| --- | --- | --- | --- | --- | --- | --- | --- |
|  |  |  | ALL (N=149) | | A (N=99) | B (N=35) | C (N=15) |
| COVID-19 vaccination prior to study enrolment | | | | | | | |
| BNT162b2/BNT162b2/BNT162b2 | 9 |  | 34 | 28 | | 5 | 1 |
| BNT162b2/BNT162b2/mRNA-1273 | 0 |  | 5 | 2 | | 0 | 3 |
| BNT162b2/mRNA-1273/BNT162b2 | 1 |  | 6 | 4 | | 2 | 0 |
| mRNA-1273/BNT162b2/BNT162b2 | 0 |  | 1 | 1 | | 0 | 0 |
| mRNA-1273/MRNA-1273/BNT162b2 | 0 |  | 1 | 0 | | 1 | 0 |
| mRNA-1273/mRNA-1273/mRNA-1273 | 0 |  | 10 | 5 | | 2 | 3 |
| ChAdOx1 nCoV-19/BNT162b2/BNT162b2 | 0 |  | 2 | 2 | | 0 | 0 |
| ChAdOx1 nCoV-19/mRNA-1273/mRNA-1273 | 0 |  | 1 | 1 | | 0 | 0 |
| BNT162b2/BNT162b2 | 11 |  | 30 | 19 | | 8 | 3 |
| BNT162b2/mRNA-1273 | 0 |  | 11 | 5 | | 6 | 0 |
| mRNA-1273/mRNA-1273 | 2 |  | 8 | 4 | | 4 | 0 |
| mRNA-1273/BNT162b2 | 1 |  | 0 | 0 | | 0 | 0 |
| ChAdOx1 nCoV-19/mRNA-1273 | 0 |  | 1 | 0 | | 0 | 1 |
| ChAdOx1 nCoV-19/BNT162b2 | 0 |  | 1 | 1 | | 0 | 0 |
| ChAdOx1 nCoV-19/ChAdOx1 nCoV-19 | 1 |  | 1 | 1 | | 0 | 0 |
| BNT162b2 | 2 |  | 13 | 11 | | 1 | 1 |
| mRNA-1273 | 0 |  | 2 | 0 | | 2 | 0 |
| ChAdOx1 nCoV-19 | 0 |  | 1 | 1 | | 0 | 0 |

**Table S2.** Known molecular diagnoses of participants with inborn error of immunity

| Subgroup A – Primary antibody deficiency  n = 99 | Subgroup B – Combined immunodeficiency  n = 35 | Subgroup C – Other inborn error of immunity  n = 15 |
| --- | --- | --- |
| *AICDA* *12p13* variant (1, 1.01%)  16p11,2-12,2 deletion *CD19* and *LAT* (1, 1.101%)  *CARD11* GOF (1, 1.01%)  *UNC13D* c.1389+1G>A, p.(?) (1, 1.01%)  *CHD7* c.3224A>G, p.(Tyr1075Cys) (1, 1.01%)  *PIK3CD* c.39C>G, p.(Thr13=) (1, 1.01%)  *NFKB1* c.482_483insCC, p.(Cys162Argfs*3) (1, 1.01%) | STAT3 deficiency (1, 2.85%)  PNP deficiency *His257 ASP* (1, 2.85%)  *TTC7A* variant, (1, 2.85%)  *CD79A* c.419C>A, p.(Thr140Asn) (1, 2.85%)  *RAC2* *c.202C>T, p.(Arg68Trp)* (1, 285%)  *ZAP-70* variant (1, 2.85%)  *ICOSL-G* variant (1, 2.85%)  22q11 deletion syndrome (3, 8.57%)  *DOCK2* variant (1, 2.85%) | STAT1 deficiency (2, 13.33%)  *NCF1* c.123A>G, p.(Lys41Arg)  CARD9 deficiency (1, 6.66%)  *CTLA-4*  c,406C>T, p,Pro136Ser (1, 6.66%)  *CTLA-4* *c.208C>T p.Arg70Trp* (1, 6.66%)  *CTLA-4* c.326 G>A (p.Gly109Glu) (1, 6.66%)  *IL-17RC* variant (1, 6.66%) |

**Table S3. Geometric mean ratio of serological responses.** Abbreviations: R, receptor binding protein; S, spike protein; N or NP, nucleocapsid protein; **IEI,** inborn error of immunity. *indicates **p< 0.05**

| Geometric mean ratio | Healthy participants | IEI participants | p-value |
| --- | --- | --- | --- |
| Response Ratio at 4 weeks after completing primary series (2 doses for healthy and 3 doses for IEI) | | | |
| Anti-RBD IgG | 5.32 [3.71–7.61] | 5.19 [3.64–7.39] | 0.93 |
| Anti-S IgG | 3.13 [2.05–4.77] | 6.17 [4.13–9.22] | 0.02* |
| Anti-N IgG | 1.04 [0.91–1.19] | 1.31 [1.13–1.52] | 0.02* |
| Decaying Ratio at 24 weeks after completing primary series (2 doses for healthy and 3 doses for IEI) | | | |
| Anti-RBD IgG | 0.34 [0.15–0.77] | 0.77 [0.29–2.03] | 0.24 |
| Anti-S IgG | 0.57 [0.22–1.49] | 0.92 [0.39–2.17] | 0.54 |
| Anti-N IgG | 0.94 [0.62–1.43] | 2.36 [1.31–4.27] | 0.02* |

**Table S4. COVID-19 infection events recorded prior and during the study.** Abbreviations: n, Number of healthy or immunodeficient participants; mABs, monoclonal antibodies; D1, before dose 1; D2, before dose 2; D3, before dose 3; D3-24, 24 weeks after dose 3.

|  | Healthy (n= 37) | IEI (n=149) |
| --- | --- | --- |
| Number of Events, n | 31 | 42 |
| Number of Patients, n (%) | 24 (64.86) | 40 (26.85) |
| Severity, n (%) | | |
| Mild – no medical intervention | 9 (29.03) | 13 (30.95) |
| Moderate – minimal intervention (medication, medically attended) | 1 (3.23) | 20 (47.62) |
| Severe – Hospitalization | 0 | 1 (2.38) |
| Serious – Life Threatening | 0 | 0 |
| Death – fatal | 0 | 0 |
| Missing Information | 21 (67.74) | 8 (19.05) |
| Infection events by period | | |
| Mar 2020 – Dec 2020 | 9 (29.03) | 0 |
| Jan 2021 – May 2021 | 6 (19.35) | 1(2.38) |
| Jun 2021 – Dec 2021 | 1 (3.23) | 6 (14.29) |
| Jan 2022 – Mar 2022 | 5 (16.13) | 23 (54.76) |
| Apr 2022 – Jun 2022 | 6 (19.35) | 12 (28.57) |
| Jul 2022 – end of the study | 4 (12.90) | 0 |
| Infection events by vaccination | | |
| Before D1 | 15 (48.39) | 1 (2.38) |
| Between D1 and D2 | 0 | 0 |
| Between D2 and D3 | 5 (16.13) | 9 (21.43) |
| Between D3 and D3-24 | 11 (35.48) | 32 (76.19) |
| Treatment received | | |
| Nirmatrelvir/Ritonavir | 0 | 8 (19.05) |
| Remdesivir | 0 | 1 (2.38) |
| Sotrovimab | 0 | 8 (19.05) |
| Tocilizumab | 0 | 0 |
| Dexamethasone | 0 | 0 |
| No treatment ^a^ | 9 (29.03) | 19 (45.24) |
| Missing information | 22 (70.97) | 8 (19.05) |

^a^ No treatment of antivirals or mABs listed in this table was given for the reported infections, however; other types of treatments may have been used but not recorded.

**Table S5.** Medically attended adverse events unrelated to SARS-CoV-2 infections

|  | Healthy  (n= 37) | Immunodeficient  (n= 149) |
| --- | --- | --- |
| Number of Events, n | 1 | 21 |
| Number of Patients, n (%) | 1 (2.7) | 17 (11.4) |
| Severity*, n (%) | | |
| Mild | 0 | 0 |
| Moderate | 0 | 19 (90.5) |
| Severe | 0 | 2 (9.5) |
| Serious | 1 (100) | 0 |
| Type of care sought or received (n, %) | | |
| Telemedicine | 0 | 7 (33.3) |
| In-person clinic | 1 (100.0) | 9 (42.9) |
| Emergency Department | 0 | 2 (9.5) |
| Hospitalization | 0 | 2 (9.5) |
| COVID assessment center | 0 | 0 |
| Health Line | 0 | 0 |
| Nurse | 0 | 1 (4.8) |
| Pharmacist | 0 | 0 |
| Physiotherapy | 0 | 0 |
| Other | 0 | 0 |
| List of AE (most common to least common) (n, %) | | |
| Miscarriage | 1 (100.0) | 0 |
| Pneumonia | 0 | 3 (14.3) |
| Upper Respiratory Infection | 0 | 3 (14.3) |
| Sinus Infection | 0 | 2 (9.5) |
| Lymphadenitis | 0 | 2 (9.5) |
| Blepharitis | 0 | 2 (9.5) |
| UTI | 0 | 1 (4.8) |
| Infected Tooth | 0 | 1 (4.8) |
| Otitis Media | 0 | 1 (4.8) |
| Pulmonary aspergillosis | 0 | 1 (4.8) |
| Lower Respiratory Infection (possible bronchitis) | 0 | 1 (4.8) |
| COPD exacerbation | 0 | 1 (4.8) |
| Neutropenia <1.7 | 0 | 1(4.8) |
| Post-polypectomy bleeding | 0 | 1 (4.8) |
| Hair loss | 0 | 1 (4.8) |
| Average (mean) onset of AE from vaccination (days, SD) | 13(0) | 88.3 (70.6) |
| Adverse events by vaccination (n, events per total # of participants observed during that period) | | |
| Between D1 and D2 | 1 (1/8) | 0 (0/20) |
| Between D2 and D3 | 0 | 0 (0/60) |
| Between D3 and D3-24 | 0 | 21 (21/112) |
| Adverse events by vaccine dose (n, %) | | |
| Between D1 and D2 | 1 (100.0) | 0 |
| Between D2 and D3 | 0 | 0 |
| Between D3 and D3-24 | 0 | 21 (100.0) |
| Between D3 and D3-4 | 0 | 7 (33.3) |
| Between D3-4 and D3-24 | 0 | 14 (66.7) |
| Adverse Events Relatedness (n, %) | | |
| Definite | 0 | 0 |
| Probably | 0 | 1 (4.8) |
| Possible | 1 (100.0) | 2 (9.5) |
| Unlikely | 0 | 1 (4.8) |
| Unrelated | 0 | 17 (81.0) |

***Severity of SARs-CoV2 adverse events was defined as follows:** mild – adverse event not requiring any medical attention; moderate – medically attended adverse event requiring minimal intervention (e.g. medication); severe – medically attended adverse event requiring hospitalization; serious – medically attended adverse event that was life-threatening

**3. FIGURES**

**Figure S1**.

**Figure S2**. Summary of participant recruitment (n*)* and number of participant visits at each timepoint (V). Abbreviations: D1, before dose 1; D2, before dose 2; D2+4wk, 4 weeks after dose 2; D2+24wk, 24 weeks after dose 2; D2+48wk, 48 weeks after dose 2; D3, before dose 3; D3+4wk, 4 weeks after dose 3; D3+24wk, 24 weeks after dose 3


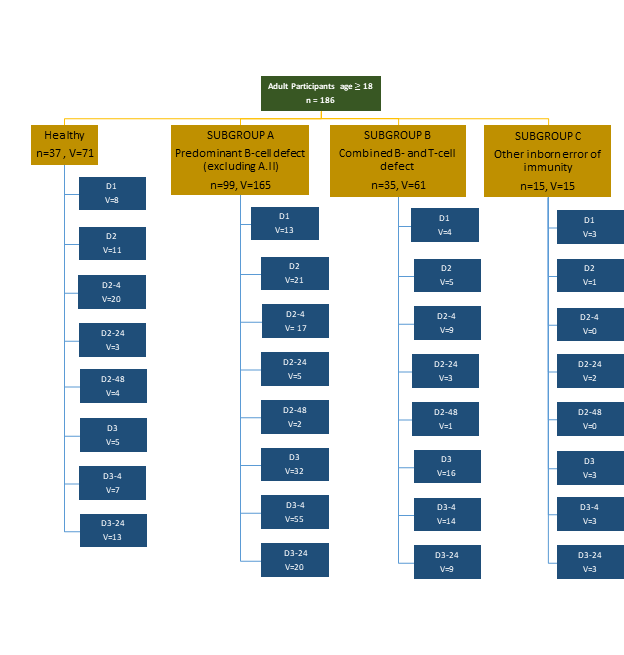


**Figure S3. Anti-S IgG responses by participant sex**


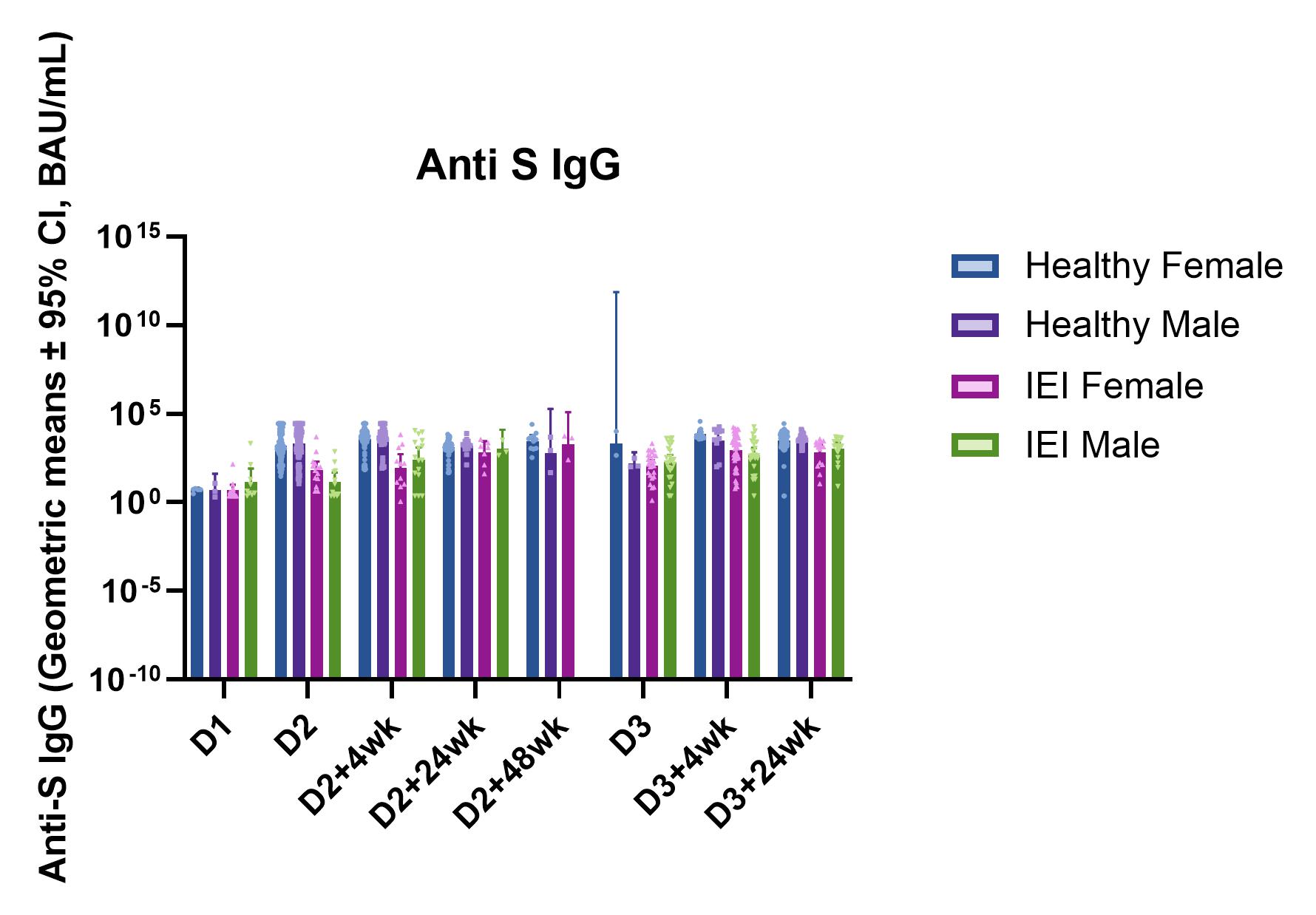


**Figure S4.** A) Baseline serum IgA levels by healthy and IEI subgroups PAD, CID, and III. B) Geometric mean anti-S IgA serology of healthy participants as well as participants with immunodeficiency with and without low baseline IgA. * indicates p <0.05, ** indicates p < 0.01, ***indicates p < 0.001, **** indicates p <0.0001, ns indicates not significant. Error bars indicate the 95% CI.

**A)**

**B)**


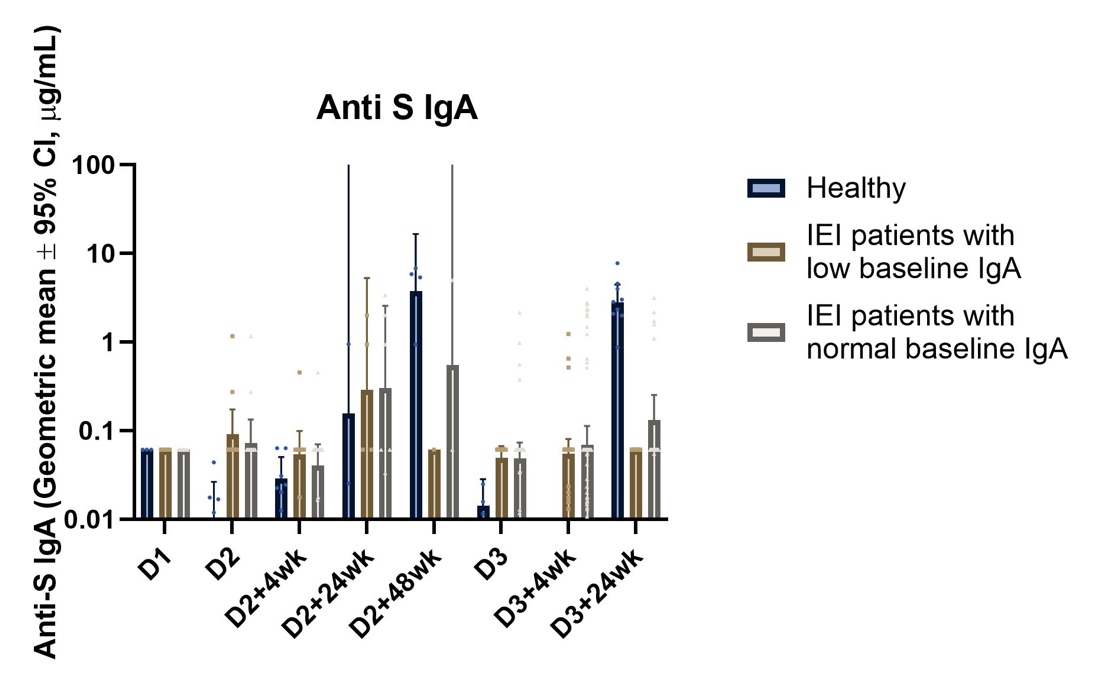


**Figure S5. Anti-S and anti-N IgG titers by blood draw date in patients with x-linked agammaglobulinemia who are on IGRT.** A) anti-S IgG titres and B) anti-N IgG titres in patients with x-linked agammaglobulinemia who are receiving IGRT.

**A)**

**B)**

**Figure S6.** Geometric mean serology of anti-N immunoglobulin titers among inborn error of immunity participants receiving and not receiving immunoglobulin replacement therapy at different timepoints pre- and post-COVID-19 vaccination up to 24 weeks after dose 3. Results include data analyzing A) anti-N IgG, B) anti-N IgA, and C) anti-N IgM. * indicates p <0.05, ** indicates p < 0.01, ***indicates p < 0.001, **** indicates p <0.0001, ns indicates not significant. Error bars indicate the 95% CI. Abbreviations: IGRT, immunoglobulin replacement therapy; CI, confidence interval


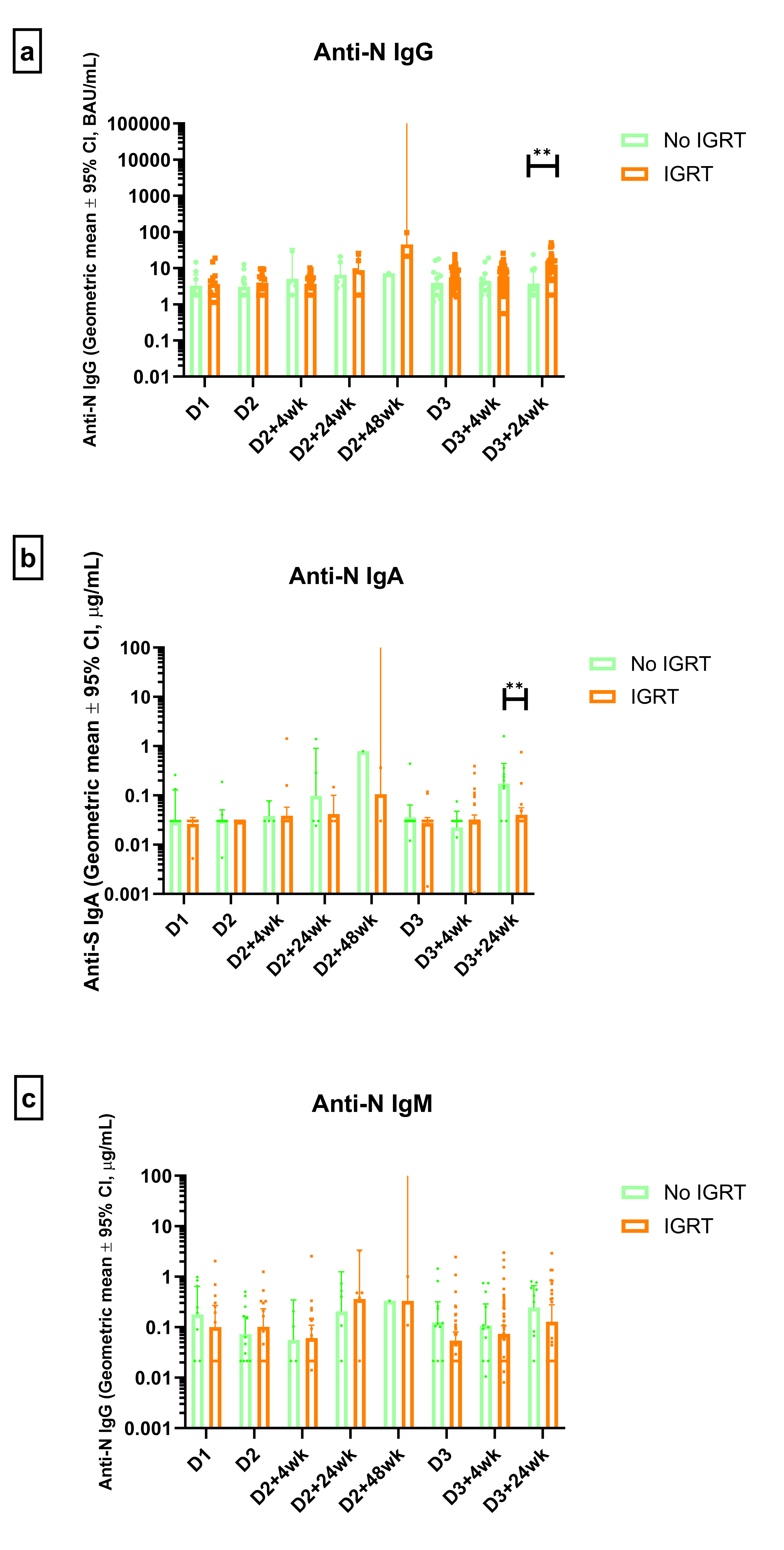


**Figure S7. Anti-S IgG and IgA among participants with primary antibody deficiency receiving and not receiving IGRT.** Geometric mean serology of A) anti-S IgG and B) anti-S IgA among primary immunodeficient participants with predominant B cell immunodeficiency (PAD subgroup) receiving and not receiving immunoglobulin replacement therapy at different timepoints pre and post-COVID-19 vaccination up to 24 weeks after dose 3. * indicates p <0.05. Error bars indicate the 95% CI. Abbreviations: IGRT, immunoglobulin replacement therapy; CI, confidence interval

**A)**

**B)**

**Figure S8. Correlation matrices of serological and cellular responses after Doses 2 and 3 among participants with inborn error of immunity and healthy controls.** Matrices are organized by participant group and timepoint of blood draw for serological and cellular analysis: A) 4 weeks after dose 2; B) 4 weeks after dose 3; C) 24 weeks after Dose 3; Matrices on the left panel represents data of healthy controls, and on the right panel of IEI participants. Abbreviations: D2+4wk, 4 weeks after dose 2; D3+4wk, 4 weeks after dose 3; D3+24wk, 24 weeks after dose 3; R, receptor binding protein; S, spike protein; N or NP, nucleocapsid protein; ID50, infective dose 50; IEI, inborn errors of immunity.


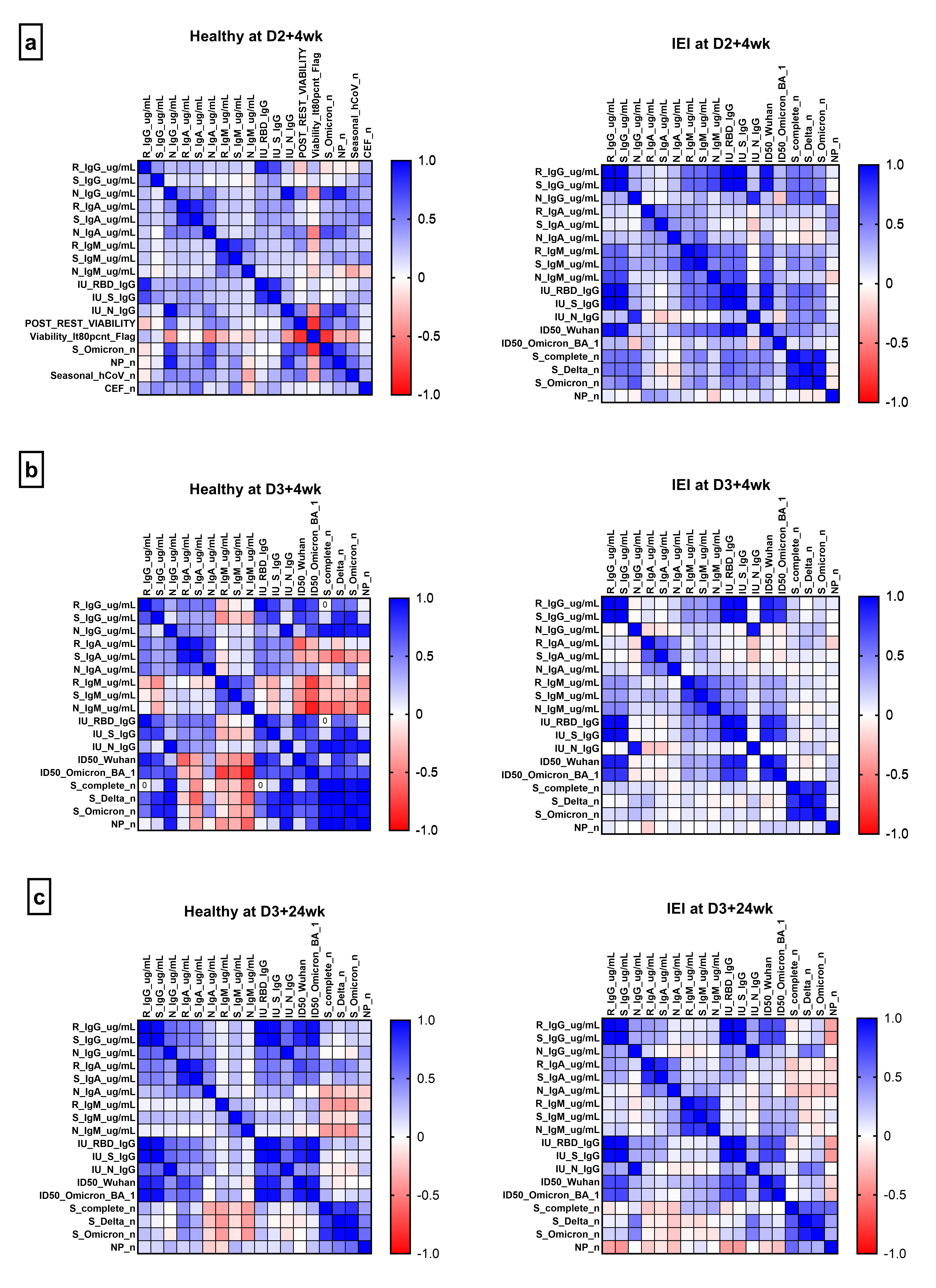

Supplement: Supplementary file 1 [file DataSheet1.docx]
